# Web-Based Technologies to Support Carers of People Living With Dementia: Protocol for a Mixed Methods Stepped-Wedge Cluster Randomized Controlled Trial

**DOI:** 10.2196/33023

**Published:** 2022-05-19

**Authors:** Clare Wilding, Debra Morgan, Jennene Greenhill, David Perkins, Megan E O'Connell, Michael Bauer, Jane Farmer, Catherine Morley, Irene Blackberry

**Affiliations:** 1 John Richards Centre for Rural Ageing Research La Trobe Rural Health School La Trobe University Wodonga Australia; 2 Canadian Centre for Health and Safety in Agriculture University of Saskatchewan Saskatoon, SK Canada; 3 Faculty of Health Southern Cross University Bilinga Australia; 4 College of Medicine and Public Health Flinders Medical Centre Flinders University Renmark Australia; 5 Centre for Rural and Remote Mental Health The University of Newcastle Orange Australia; 6 Health Research Institute University of Canberra Canberra Australia; 7 Department of Psychology University of Saskatchewan Saskatoon, SK Canada; 8 Australian Centre for Evidence Based Aged Care La Trobe University Melbourne Australia; 9 Social Innovation Research Institute Swinburne University of Technology Melbourne Australia; 10 Wimmera Health Care Group Horsham Australia

**Keywords:** virtual, dementia, community, rural, carer, caregiver, mobile phone

## Abstract

**Background:**

Informal carers play a significant role in supporting people living with dementia; however, carers in rural areas are often isolated, with limited access to support services. Although dementia-friendly communities provide valued support for carers, access to them is limited as they are few and geographically dispersed.

**Objective:**

This study’s aim was to increase support and services for rural informal carers of people living with dementia by using information and communication technologies accessed through an integrated website and mobile app—the Verily Connect app. The objective of this protocol is to detail the research design used in a complex study that was situated in a challenging real-world setting integrating web-based and on-ground technology and communication. Therefore, it is anticipated that this protocol will strengthen the research of others exploring similar complex concepts.

**Methods:**

A stepped-wedge, open-cohort cluster randomized controlled trial was conducted to implement Verily Connect across 12 rural Australian communities. The Verily Connect intervention delivered web-based, curated information about dementia, a localized directory of dementia services and support, group and individual chat forums, and peer support through videoconference. During the implementation phase of 32 weeks, Verily Connect was progressively implemented in four 8-weekly waves of 3 communities per wave. Usual care, used as a comparator, was available to carers throughout the study period. Participants and researchers were unblinded to the intervention. There were 3 cohorts of participants: carers, volunteers, and staff; participants were recruited from their communities. The primary outcome measure was perceived carer social support measured using the Medical Outcomes Study-Social Support Survey. Volunteers and staff provided feedback on their participation in Verily Connect as qualitative data. Qualitative data were collected from all cohorts of participants through interviews and focus groups. Process evaluation data were collected through interviews and memos written by research staff. Data on the costs of implementing Verily Connect were collected by the research team members and evaluated by a health economist.

**Results:**

Between August 2018 and September 2019, a total of 113 participants were recruited. There were 37 (32.7%) carers, 39 (34.5%) volunteers, and 37 (32.7%) health service staff. The study was complex because of the involvement of multiple and varied communities of carers, volunteers, health service staff, and research team members originating from 5 universities. Web-based technologies were used as intervention strategies to support carers and facilitate the process of undertaking the study.

**Conclusions:**

The Verily Connect trial enabled the testing and further development of a web-based approach to increasing support for carers of people living with dementia across a diverse rural landscape in Australia. This protocol provides an example of how to conduct a pragmatic evaluation of a complex and co-designed intervention involving multiple stakeholders.

**Trial Registration:**

Australian New Zealand Clinical Trials Registry ACTRN12618001213235; https://tinyurl.com/4rjvrasf

**International Registered Report Identifier (IRRID):**

RR1-10.2196/33023

## Introduction

### Background

Approximately 50 million people live with dementia worldwide [1], including an estimated 470,000 Australians [2]. Much of the care given to people living with dementia is provided by informal carers, including family, friends, and neighbors, who do so without financial remuneration [3,4]. Although caring is frequently motivated by love and concern, caring for someone with dementia can also be burdensome and is linked with the added risk of social isolation [5-7] and carer distress [8]. Those who care for a person living with dementia tend to have less time for holidays, leisure activities, and family and friends [9]. As a group, carers are more likely to have smaller social networks than people without caring responsibilities, and social support can often decrease over time [7,10]. Although informal carers are at risk of social isolation, when social support is available, social isolation is reduced [7], which points to the need to find ways to increase formal and informal social support for carers.

The health of people living in rural and remote areas of Australia is noted to be poorer than that of people living in metropolitan areas, and access to health services is also reduced in rural and remote areas [11]. In rural areas, obtaining support for informal carers of people living with dementia is very challenging as rural people must often travel long distances to access specialist services [12]. In addition, rural dementia service users, carers, and providers report challenges and vexation when trying to locate appropriate services within a fragmented health system [13]. Dementia often attracts social stigma [14] and, in small rural communities in which residents are familiar with each other, carers of people living with dementia may avoid seeking support to maintain their privacy.

Although the peak organization for providing dementia information and support in Australia, Dementia Australia, provides a range of resources for people living with dementia and carers, there remain challenges for rural Australians in accessing resources relevant to their local area context [15]. For example, some services (such as carer support groups and dementia cafés) are not available in most rural communities. The Dementia Friendly Community movement, which aims to help members of communities better understand dementia and be aware of and accept people living with dementia in their community [16], helps address challenges in supporting rural carers. However, the support provided is solely for communities that have invested in becoming a dementia-friendly community. Thus, access to this support can be patchy. For rural people, the challenge is not lack of existence of services and support per se but rather that programs are provided distant from where rural people live [15].

The Verily Connect study was built on the hypothesis that information and communication technologies (ICTs) could be leveraged to provide increased support and services to rural carers of people living with dementia. (The study is named as a loose acronym for Virtual Dementia-Friendly Rural Community.) Using technology, rural carers might be assisted to identify, contact, and find their way to the services and support they need using easily accessible and locally relevant information anytime and on demand provided by the specially designed Verily Connect app. Along with the app, the use of videoconferencing to facilitate peer support and information sharing between carers was an additional feature designed to help reduce the need to travel to access support. Videoconferencing support groups for carers of people living with dementia have been demonstrated to reduce caregiver distress, depression, and feelings of loneliness [17]. The Verily Connect project was hypothesized to provide a way to build a web-based dementia-friendly community that would also be connected to and support on-the-ground dementia-friendly communities. Provision of support on the web (through information and web-based peer support) was proposed to augment the support available to the on-the-ground communities in an efficient and cost-effective way and thus assist with scaling up the spread of on-the-ground dementia-friendly rural communities.

### Prior Work

Although there has been significant uptake in work using web-based technologies to support carers of people living with dementia within the last 5 years (and especially in response to COVID-19 pandemic social distancing restrictions), the idea was novel in 2016, when the Verily Connect study was pitched for funding. A systematic review of studies using web-based technology to support informal carers of people living with dementia identified only 3 relevant studies in the period of 1990 to 2012 [18]. In 2013, Godwin et al [18] concluded that, although feasibility studies had shown promising results for using web-based technology to reduce the burden experienced by informal carers, more randomized controlled trials were needed to evaluate these interventions. Another systematic review of internet-based supportive interventions for carers of people living with dementia conducted in 2014 concluded that, although interventions that combined information and opportunities for peer interaction showed the most potential, more research on these types of interventions was needed [19]. Several studies evaluating the use of web-based technology to support carers of people living with dementia were commenced in 2016 to 2017 [20-22].

The Verily Connect project was designed to build on and extend the previous work of project investigators and other researchers. In 2016, a study was completed in rural communities in Australia to identify gaps in service provision and support for people living with dementia and their carers [23]. This research found that carers of people living with dementia in the community would benefit from learning about services that were available in their local geographical area and how they could access these services; having someone to talk with and the opportunity to receive respite were also reported as service gaps [23]. A finding of the study was that local service delivery might be improved by training volunteers to work in an integrated way with health care providers to assist and support carers [23].

On the basis of the project findings, a prototype smartphone app for helping carers of people living with dementia navigate health and aged care services and increasing support and connection between carers and service providers was piloted in 2016 with 2 rural communities. The Service Navigation and Networking for Dementia in Rural Communities app was co-designed and coproduced with rural carers, service providers, and other stakeholder representatives (including Alzheimer’s Australia Victoria) [24]. Feedback from the pilot indicated that more flexible support from services was needed and that carers (most of whom were older people) needed assistance in developing confidence in using web-based technologies.

The videoconferencing aspect of this study was based on the work of O’Connell et al [25] in rural Canada. That study demonstrated that support groups conducted by videoconference were able to increase support for people living with dementia and their carers. This type of technology-enabled service was advantageous for the rural participants as it reduced the need to travel long distances in sometimes treacherous weather and road conditions to access the needed support.

### Goal of the Study

The Virtual Dementia-Friendly Rural Communities (Verily Connect) project aimed to develop, trial, and evaluate a web-based intervention for increasing support for informal carers of people living with dementia in rural communities. The main objective was to determine whether support perceived by carers, as measured by the Medical Outcomes Study-Social Support Survey (MOS-SSS), increased after the implementation of the Verily Connect intervention.

A secondary objective was gaining feedback about the usability and usefulness of Verily Connect. Another secondary objective was to evaluate the process of implementing Verily Connect to better understand the barriers to and enablers of its implementation and to complete a cost analysis of implementing Verily Connect.

## Methods

### Study Design

A stepped-wedge, open-cohort cluster randomized controlled trial design [26,27] was used to trial the implementation of Verily Connect across 12 rural Australian communities. The trial was registered with the Australian New Zealand Clinical Trials Registry (ACTRN12618001213235). Each cluster comprised 1 geographically defined rural community. During the control period, the participants had access to their usual care. During the implementation period (32 weeks in total), Verily Connect was progressively implemented in each of the 12 participating rural communities across 4 periods of 8 weeks each. (Each 8-week period was considered a *wave*.) In each wave, 3 clusters moved from the control phase to the implementation phase. Thus, at 8-weekly intervals, 3 additional clusters (ie, 3 rural communities) received the Verily Connect implementation. Multimedia Appendix 1 provides a diagram of the stepped-wedge control and implementation periods.

Each cluster experienced a control phase and an intervention phase. However, the length of the control and intervention phases differed for each cluster. Some clusters commenced the intervention earlier and had a longer exposure than clusters that commenced later. A staggered start date for different clusters was pragmatic; start-up activities such as volunteer training and onboarding of participants to use the technology were significant and, therefore, each wave needed an 8-week implementation cycle. However, owing to funding requirements, the entire project (from ethics application to final reporting) needed to be fully executed within 3 years. The implementation phase commenced in August 2018 and concluded in March 2019.

### Ethics Approval

The study received approval from the Melbourne Health Human Research Ethics Committee (application HREC/17/MH/404, reference 2017.376) after being assessed as meeting the requirements of the Australian National Statement on Ethical Conduct in Human Research (2007) and of the Helsinki Declaration of 1975, as revised in 2000.

### Intervention

There were 3 main components of the Verily Connect intervention. Two of these components were web-based, and the third was designed to provide carer participants with additional support in using the web-based components. The 3 components are outlined in Textbox 1.

**The 3 main components of the Verily Connect intervention.**

**Intervention components**

1. An integrated website and mobile app (Verily Connect app). The Verily Connect app had 2 main functions: information provision and facilitation of social communication between users. General information relevant to a carer of a person living with dementia was provided by 12 *guides*. The guides were developed by the research team; they were deliberately brief and curated from freely accessible but reputable internet sources. Links to information sources were accessible on the app and could be clicked to open the linked source in a web browser. For each participating community, there was a directory of locally available dementia-relevant services that were geographically displayed using Google Maps. Service information included links within the app that directly connected app users to the telephone, email, Facebook, and website of the listed service (where available). In addition, the Verily Connect app provided opportunities for app users to connect with each other using a text-based chat function presented as *forums*. During the trial, to control when communities had access to the Verily Connect intervention, access to the Verily Connect app was password-protected; the research team gave participants access to a password and the app when their community entered the intervention phase.
2. Carer peer support groups that met via Zoom (Zoom Video Communications) videoconference. The project manager facilitated the implementation of the support groups by providing technical assistance and information about the group and videoconference etiquette (including precautions about privacy, confidentiality, and being secure when using the internet), making introductions, and ensuring that all members were given the opportunity to contribute to discussions. Most groups did not have a specific agenda; rather, the participants could speak about whatever they wanted. The project manager used minimal questioning and prompting to encourage conversation and ensure that every participant had a turn to speak. A challenge sometimes arose if a participant had poor internet connectivity and, therefore, had trouble keeping up with the group conversation. Another challenge arose when only one of the group members joined by telephone while the others were on videoconference; the person on the telephone missed nonverbal cues provided by those on videoconference and, consequently, there were some miscommunications and frustration with mistiming of discussions. The first carer peer support group was held at the end of wave 2 as this length of time was needed for sufficient carers to be recruited and their communities to enter the intervention phase. Thereafter, the carer peer support groups met monthly. Attendance at carer peer support groups was managed by direct invitation to participants whose communities had entered the intervention phase.
3. Volunteer support and a *Technology Learning Centre* (also known as a Verily Connect Hub) that was physically located in each community. The role of the volunteers was to assist carers and other interested community members in learning how to use the Verily Connect app and other relevant web-based technologies (such as Zoom videoconferencing). Volunteers were governed by a health service or volunteer organization in their local community, and they received a day’s training from Verily Connect project staff. The Verily Connect project also facilitated support for volunteers via group videoconference meetings. Verily Connect Hubs were slightly different in each community; however, each community was given an iPad (Apple Inc) and Samsung S4 phone and an Aus $2000 (US $1419.60) budget to purchase resources for the Hub, such as books about dementia, web cameras, headsets, tablets, and items to assist people living with dementia (eg, simplified clocks, therapy dolls, and activities for people living with dementia). The Hubs were established, and volunteers received their training only when the community entered the intervention phase.

The Verily Connect app was developed and published on the web before the first intervention period wave. Selected screenshots of the Verily Connect app are provided in Multimedia Appendix 2. There were no substantial revisions or updates to the Verily Connect intervention during the trial. Carer participants were requested to use the Verily Connect app at least 4 times; however, they could decide how much or how little time they spent on each opening of the app. Carer participants were also asked to take part in at least one videoconferenced peer support group.

### Comparators

During the control phase, informal carers and people living with dementia received their usual care and support, which differed in each community. Most communities did not offer specialist dementia support; they offered only general health care. However, two of the communities provided a specialist dementia support nurse on a part-time basis. In six of the communities, no carer support groups were operating. In 4 communities, general carer groups were available and, in 2 communities, dementia-specific carer support groups were offered. A usual practice comparator is considered the most appropriate approach for complex, nonpharmacologic interventions such as Verily Connect because other types of comparators are unfeasible [28]. When a community entered the intervention phase, they received usual care and support with the addition of the Verily Connect intervention strategies. For each of the 12 communities, the control phase was an 8-week preintervention wait period followed by the sequential stepped crossover to commence the Verily Connect intervention phase. All participating communities eventually received the Verily Connect intervention.

### Study Setting and Participants

The study population comprised people in 12 rural communities in 3 Australian states (8/12, 67% in Victoria and 2/12, 17% in both New South Wales and South Australia). In each rural community, there were representatives from 3 subpopulations: service providers (*staff*), volunteers (*volunteers*), and informal carers of people living with dementia (*carers*). Having participants from 3 different stakeholder groups ensured that data on the effects of Verily Connect could be collected from each group’s perspective.

### Project Team

The project team included research officers, a project manager, a steering committee, and an advisory committee. A research officer was assigned to 2 or 3 clusters to coordinate project activities, promotion, participant recruitment, data collection, and regular engagement and contact with participants, including traveling to the communities as needed. A research project manager oversaw the operations of the research officers, presented at community meetings, facilitated videoconference meetings with groups of participants, and liaised with other stakeholders. A research steering committee met monthly to monitor the overall design and progress of the study. An advisory committee met quarterly to provide input about the project from the perspective of app end users and participants; it comprised carers of people living with dementia and representatives from service organizations that supported people living with dementia and carers.

### Randomization

The different participant groups had different roles during the Verily Connect intervention, which precluded randomization of individuals; however, it was possible to randomize the order in which the participating communities joined the intervention phase of the study. An independent consultant used stratified randomization to produce the schedule. Stratification was needed to ensure that only 1 community from the states of South Australia and New South Wales began in a wave as there was only 1 part-time research officer allocated for each of these states. It would have been impractical for 2 communities managed by the same research officer to commence the intervention within the same wave. The sequence in which communities would join the intervention phase was revealed to the project manager before the start of the trial so that the project manager could organize the logistics of implementing the intervention in the communities. The communities (including the participants) progressively learned the order of implementation immediately before the beginning of the next wave of intervention implementation. For example, at the beginning of wave 1, only the 3 communities that would commence the intervention in wave 1 were known. At the beginning of wave 2, the identities of the next 3 communities joining the intervention phase were revealed.

### Inclusion Criteria

The Verily Connect intervention was designed to take action at the community level and, therefore, the parameters for inclusion were broad. Although it was anticipated that many carers would be aged >75 years as dementia disproportionately affects older people and it was anticipated that many carers would be spouses of people living with dementia, there were no specific age range requirements for carers other than being an adult (aged ≥18 years). In addition, the stage of dementia of the person living with dementia was not specified; all carers of people living with dementia were welcome to participate, including carers of people who were living with mild cognitive impairment. Although it is acknowledged that the needs of carers of people living with mild cognitive impairment and those living with dementia are different, both groups of people could elect to participate if they chose to as the study was a practical trial designed to intervene at the community level rather than solely targeting individuals. Allowing carers of people living with mild cognitive impairment was in keeping with the broad and inclusive intent of the project. The aim was to increase social support for carers no matter the stage of dementia of the person for whom they were providing care. The inclusion criteria for carers, volunteers, and staff are outlined in Textbox 2.

**Inclusion criteria for carers, volunteers, and staff.**

**Inclusion criteria for carers**

- Self-identified carer for a person living with dementia or cognitive impairment
- Living in the community catchment area
- Willing to try the Verily Connect app on a smartphone or tablet with internet access or to use the website on a computer with internet access and willing to participate in peer support groups via videoconference on an electronic communication device with internet access and videoconferencing functionality

**Inclusion criteria for volunteers**

- Living in the community catchment area
- Willing to undertake training provided through the Verily Connect project
- Willing to assist people to learn to use web-based technologies

**Inclusion criteria for staff**

- Employed by a provider of a dementia service or service for older adults in the catchment area
- Access to a smartphone, tablet, or computer with internet access

### Recruitment

Recruitment began 8 weeks before the start date for wave 1 of the implementation phase and continued until the end of wave 4. Recruitment was undertaken by conducting open community forums in each of the 12 participating communities. Additional recruitment strategies included meetings with community groups (eg, Lions Club, Probus, carers’ support groups, and Country Women’s Association), promotion via social media (Twitter and Facebook) and websites of partner health services, and press releases and advertising using news media (radio and print). Participant information and consent forms (Multimedia Appendix 3) were available at the recruitment meetings and provided to all potential participants. Potential participants were given the opportunity to discuss their participation with and ask questions to a research officer or the project manager; discussions were available face to face and by telephone. After the participants provided their written consent, the research officer assigned to the participant’s community enrolled the participant.

### Sample Size and Power

The sample size was based on an estimation of how many carers of people with dementia might be available to participate from a small rural community, the number of volunteers required to support the carers, and the number of health service staff who might have expertise in supporting people with dementia in a small rural community. At the time of the protocol development, there were no suitable studies to inform our sample size calculation. Therefore, a linear mixed effects model simulation (2000 replications), where the intervention and period effects were assumed to be fixed and the carer and community effects were assumed to be a random simulation, was used instead. The simulations were run in R (version 3.51; R Foundation for Statistical Computing). The assumptions were 12 rural communities with 12 carers from each community that contribute data at each of the 5 data collection periods, a difference in the mean MOS-SSS score of 9 [29] between the intervention and control phases, an SD of 24.2, an α of .05 for a 2-sided test, an intracommunity correlation coefficient of 0.01 and 0.05, and within-carer correlation (0.3, 0.5, and 0.7) for repeat outcome measures on carers (Table 1).

The study power was calculated as the proportion among all 2000 simulation runs of 2-sided *P* values for the estimated intervention effect that reached a nominal value of <.05. A total of 2000 replications were sufficient to estimate the power with a margin of error of 1.75% assuming the true power was 80% [30]. To allow for a dropout rate of 20%, recruitment targets were set per community at 15 carers, 3 health service staff, and 5 volunteers.

**Table 1:** Power calculations to detect an effect size of 9 (SD 24.2) between the intervention and control phases assuming an alpha of 5% for a 2-sided test for a stepped-wedge cluster randomized controlled trial with 12 clusters and 5 steps (including baseline).

Within-carer correlation	Within-community correlation	Sample cluster size^a^	Power^b^
0.3	0.01	12	0.80
0.5	0.01	12	0.89
0.7	0.01	12	0.98
0.3	0.05	12	0.78
0.5	0.05	12	0.88
0.7	0.05	12	0.97

^a^A total of 15 carers to be recruited at baseline to allow for 20% attrition over the study period.

^b^Power calculations based on 2000 simulations.

### Outcome Measures

#### Primary Outcome

The primary outcome was the change in perceived carer social support as measured by the MOS-SSS [31]. The Zarit Burden Interview (ZBI) [32] was initially selected as the primary outcome, and the MOS-SSS was selected as a secondary outcome. However, 1 month into the trial, the MOS-SSS was elevated to the primary outcome and the ZBI became a secondary outcome as it had become clearer after further consultation with a biostatistician that carer support would likely be more amenable and responsive to the Verily Connect type of intervention than carer burden would.

#### Secondary Outcomes

The secondary outcomes are outlined in Textbox 3.

**Secondary outcomes of the study.**

**Secondary outcomes**

- Additional quantitative measures collected by web-based survey:
  Perception of carer burden, measured using the Zarit Burden Interview [32]
  Self-reported use of services available in the community to support people living with dementia or cognitive impairment and their carers, measured using a purpose-designed questionnaire
  Carers’ use of information and communication technology, measured using a survey adapted from Predictors of older adults’ technology use [33]
- Feedback about the experience of participating in Verily Connect activities from the perspective of carers and volunteers
- Process evaluation using the Consolidated Framework of Implementation Research [34]
- A cost analysis of the implementation of Verily Connect undertaken by a consultant health economist

### Data Collection

#### Survey Data

Self-reported use of services in the community was developed as a purpose-designed survey as no other existing measure could be found that collected the specific data that were required. See Multimedia Appendix 4 and 5 for a copy of the survey questions.

A survey designed for older adults was used as the basis for questions about carers’ use of ICT even though carers could be adults of any age. In the planning stages of the study, it was hypothesized that most of the carers who were likely to be involved in the study would be older people who were spouses of people living with dementia. The survey asks basic questions about ICT such as whether the carer has access to internet-enabled devices and what sort of device and whether the person had recently accessed the internet and, if so, what was the purpose of using the internet (see Multimedia Appendices 4 and 5 for a copy of the survey questions). This survey was written with older people in mind; however, it does not preclude younger people, and the survey assessed access to and use of ICT in a way that did not require technical knowledge or previous experience with using ICT.

Data were collected from carer participants through web-based surveys completed at 6 times. The first 5 were within the trial period, and the final time was 6 months after the end of the trial. The researchers emailed the participants the link to complete the appropriate survey on the web, as shown in Table 2.

Survey 1 can be found in Multimedia Appendix 4. Surveys 3, 5, and 6 were the same and can be found in Multimedia Appendix 5. Surveys 2 and 3 consisted only of the MOS-SSS. The demographic information that was collected only during survey 1 was age, gender, identification as Aboriginal or Torres Strait Islander, languages other than English spoken at home, postcode, highest level of educational attainment, relationship of the carer to the person living with dementia, length of time in the caring role, and an open-ended question about what the participant considered a dementia-friendly community to be. Background information that was collected during survey 1 and surveys 3, 5, and 6 was information about whether the carer or person living with dementia had a Health Care Card, Private Health Insurance, or a Home Care Package; whether the person living with dementia had received a diagnosis of dementia; where the person living with dementia resided in relation to the carer; whether emergency care had been used in the previous 2 months; what health and community services had been used in the previous 2 months and who had organized access to these services; whether other services were needed or received; whether access to services was easy or difficult; and the rating of the dementia-friendliness of the participant’s local community.

**Table 2:** Web-based carer survey collection periods and content.

	Collection period	Survey content
Survey 1	August 20 to August 31, 2018, or when the participant first joined	MOS-SSS^a^ ZBI^b^ Initial demographic and background information Perception of social connection
Survey 2	October 15 to October 26, 2018	MOS-SSS
Survey 3	December 10 to December 21, 2018	MOS-SSS ZBI Ongoing background information Perception of social connection
Survey 4	February 4 to February 15, 2019	MOS-SSS
Survey 5	April 1 to April 12, 2019	MOS-SSS ZBI Ongoing background information Perception of social connection
Survey 6	October 7 to October 18, 2019	MOS-SSS ZBI Ongoing background information Perception of social connection

^a^MOS-SSS: Medical Outcomes Study-Social Support Survey.

^b^ZBI: Zarit Burden Interview.

#### Feedback Data

Informal feedback from all types of participants was collected and recorded as memos by the research team throughout the study period. Memos were written about ad hoc feedback on the Verily Connect app (eg, if a service was missing or a link did not work). Memos were also written after routine follow-up by the research team (eg, feedback about whether a participant had been using the Verily Connect app, if they had experienced any problems using the app or connecting to videoconference meetings, or if they had had a good experience using the app).

Formal feedback was collected via focus groups with volunteers at the end of the implementation period (April 1-12, 2019). The focus groups were conducted by videoconference. Volunteers were asked about their experience of volunteering for Verily Connect (see Multimedia Appendix 6 for the focus group questions). During the follow-up period (September 2019-October 2019), formal feedback was collected by individually interviewing all participants (see Multimedia Appendix 7 for the interview guide). The carer, volunteer, and staff participants were asked about their use of the Verily Connect app after the end of the implementation period and whether they had any additional feedback on or suggestions to improve Verily Connect.

Feedback was included as a secondary outcome measure as it provides a description of the participants’ perceptions and perspective of the Verily Connect intervention. As the Verily Connect intervention was an innovation in carer support, the collection of detailed feedback enabled a more comprehensive and nuanced understanding of the effects of the intervention to be gathered.

#### Process Data

The Consolidated Framework of Implementation Research (CFIR) is a framework of ideas for preparing for and evaluating the implementation of innovations [35]. The CFIR Interview Guide Tool [36] was used to create an interview guide (Multimedia Appendix 8) to examine the implementation of Verily Connect. Interviews with staff were completed at the end of the implementation period (April 1-12, 2019). The interviews were conducted via videoconference or telephone.

#### Economic Data

Data for the cost analysis were collected by Verily Connect project staff about the time they spent completing Verily Connect implementation activities and the resources needed to support this work (eg, office space, travel costs, and room hire).

### Data Analyses

#### Analysis of Survey Data

The demographic data, participant reports of using technology, and perceptions of social connection were descriptively analyzed. A method suggested by Hussey and Hughes [37] using a basic model-based approach for analyzing data from a cross-sectional stepped-wedge cluster randomized controlled trial was initially planned. However, owing to difficulties with recruitment, there were insufficient samples for this method to be viable; only those participants who completed the MOS-SSS and ZBI on occasions before and after receiving the Verily Connect intervention were included. Missing data were managed by first using the guidelines for handling item- and construct-level missing data as described in the manuals for scoring the assessment instruments. Next, where participants completely failed to provide data (person-level missing data), a pairwise deletion approach was adopted. The difference between pre- and postintervention results for the MOS-SSS and ZBI was tested for statistical significance using a 2-tailed paired *t* test.

#### Analysis of Qualitative Feedback

All qualitative data were collected as text (memos were already in written form, and interviews and focus groups were transcribed verbatim). The texts were imported into NVivo (QSR International); a separate NVivo project was created for each category of participant: one for carers, one for volunteers, and one for staff. Data were initially sorted into codes as described by Miles and Huberman [38] and Stanley [39]. The coding framework was derived based on questions asked of participants, such as what type of device the participants used, how often they used the Verily Connect app and for what purposes, what they thought worked well about the Verily Connect model and what did not work well, and whether they had any suggestions for improving the Verily Connect app or model. Inductive analysis and coding were also completed. Codes were inductively developed from issues that were raised by participants; for example, the participants’ experiences of caring, their preferences for receiving support, and their experiences and perceptions of using technology to receive support.

The qualitative analysis was led by an experienced qualitative researcher (CW), who established the coding framework. Research officers assisted with reviewing the data and coding. CW completed further iterative data reduction, categorization, and theming using qualitative analysis techniques described by Streubert and Carpenter [40], Silverman [41], and Braun and Clarke [42,43]. Data saturation was not used as a stopping point for data analysis; all available data were analyzed. The findings were discussed by the research team, and all team members were involved in the final reporting.

#### Analysis of Process Data

The verbatim transcripts of staff interviews were qualitatively analyzed using the CFIR framework [44]. A CFIR codebook, descriptions, and NVivo template were downloaded from the CFIR website [44]. CW led the analysis process. Research officers assisted with the coding process using the CFIR codebook, descriptions, and template. Further iterative analysis, categorization, theming, data reduction, and selection of data for reporting were completed by a small team of researchers (CW, DM, and IB). All available data were analyzed. The final findings were discussed by the research team, and all team members were involved in the final reporting.

#### Analysis of Economic Data

A health economist created a data collection template to collect and collate resource use information according to input cost classifications. The development of the template drew heavily on the cost classification scheme outlined by the World Health Organization [45]. Research officers populated a template for each of the 12 communities. Overhead cost data that were relevant to the entire study and all 12 communities were also collected: (1) website and mobile app development, (2) advertising and promotion, (3) training development, and (4) communications. The health economist used these data to estimate the resources that would be required to implement Verily Connect in a nonresearch environment.

## Results

Data collection commenced in August 2018 and concluded in September 2019. The total number of participants was 113, comprising 37 (32.7%) carers, 39 (34.5%) volunteers, and 37 (32.7%) staff members. Target numbers for volunteer and staff participants were achieved; however, the number of carer participants was lower than was hoped for. The study was complicated to implement because of the lengthy time frame, the variety of participants (carers, volunteers, and staff) who had different roles to play, the heterogeneous nature of the participating rural communities, and the fact that the research team was geographically dispersed. Differences in government, health, and community organizations and modes of operating across the 3 states of Australia added to the complexity of implementing the study. Even within states, each of the 12 communities was distinct and differed from other communities according to geography, population size and profile, and health and community support available. The research team comprised researchers from 5 universities across 3 time zones and separated by hundreds of kilometers.

Variability across the communities was managed by allowing each community some flexibility regarding the implementation of the Verily model in the local community. For example, in Victor Harbor, two of the volunteers were a couple and worked together to assist people in their community to use Verily Connect. In addition, Victor Harbor was well-supported by active face-to-face carer support groups, so some of the carers in that community elected to continue attending their local carer support groups rather than the web-based support provided by Verily Connect. In Kooweerup, the health service leveraged interest in the Verily Connect project to springboard the development of a local dementia-friendly café that offered face-to-face social support for people living with dementia and carers.

Web-based technology in the form of the Verily Connect app was used as the main method of adding to the support received by the carer participants. Other web-based ICT was used to facilitate communication between the participants and the research team and between research team members.

A study report provided to the funding body is available on the web [46]. More detailed publication of the results is anticipated to be completed by June 2022.

## Discussion

### Principal Findings

This study aimed to develop and implement the use of web-based interventions to add to the support provided to informal carers of people living with dementia in rural communities and evaluate the developed Verily Connect program by measuring changes to carers’ perceived support as measured by the MOS-SSS. The purpose of this manuscript was to detail the methods that were used to undertake this study and, therefore, the results of the evaluation of the Verily Connect program have not been presented here but are anticipated to be discussed in future publications. As illustrated in the presentation of the study methods, the study design was quite complicated—there were 3 types of participants, and each participant group had a different role to play in the study; 12 rural communities participated in the study and were geographically dispersed across 3 states of Australia; the study took place in a community setting rather than in a laboratory or health care service; and effort was made to include carers of people living with dementia in the design of the Verily Connect app, its implementation, and the research process.

The study was complex and, therefore, challenging to implement. Although a less complex study would have been easier to complete, the complexity of the study was representative of the complexity of the phenomenon being studied. Support for informal carers of people living with dementia in rural areas of Australia is heterogeneous and messy and involves multiple stakeholders. Just as the phenomenon is complex, solutions for increasing support for informal carers are also complex and, therefore, using a simpler type of research design would have run the risk of oversimplifying the phenomenon and may have resulted in poorer-quality data.

The Verily Connect model leveraged web-based ICTs to overcome challenges to service delivery in small rural communities, especially reducing the need to travel to access dementia-specific services and gathering information about local and national services in one place. Web-based technologies were also used to facilitate research teams across multiple universities and separated by geography to collaborate and work effectively together. Further web-based technologies were used to facilitate the support of and efficient sharing of information between volunteers and health services across 3 states of Australia. The use of these technologies provided a solution for assisting geographically dispersed groups of people to collaborate, share ideas, and support each other and, thus, they are equally important in the time of physical separation created by the need to reduce social contact to avoid spreading a socially transmitted virus such as COVID-19.

The use of a stepped-wedge cluster design enabled the evaluation of the Verily Connect app in a real-world setting. The rural communities that participated as clusters in the trial were diverse in terms of geographical and population profile, access to services, funding, and infrastructure. Thus, when developing and trialing the Verily Connect implementation, there was a need for the model and the evaluation to be flexible and dynamic to meet the needs of the different local populations. Thus, flexibility was built into the Verily Connect implementation model so that each community had a license to tailor the implementation to their community.

The research was undertaken using a co-design and coproduction philosophy [47,48]. Open public forums were held at the beginning of the project to gather perspectives, needs, and ideas from carers, service providers, and community members. Throughout the project, coproduction approaches were used, such as the ongoing adaptation of the Verily Connect model based on feedback collected and challenges encountered. There was engagement with key stakeholders, including rural community members and local organizations, Dementia Australia, Carers Australia, the Commonwealth, state and local governments, and rural service providers. At the conclusion of the project, preliminary findings were shared with each community.

The aim of testing the Verily Connect model in a variety of rural communities was to learn how the model could be adapted to a wide range of rural communities, anticipating that the model might be scaled up to national implementation. To this end, a toolkit was developed to enable communities to join the web-based dementia-friendly Verily Connect community and establish a local geographical dementia-friendly community [49].

### Limitations and Strengths

A limitation of the project was the small number of carer participants in each cluster. Recruitment of carer participants was very challenging. Potential participants were dissuaded from taking part because of feeling overwhelmed with caring responsibilities and lack of interest or confidence in using web-based technology. The time and effort needed for recruitment and onboarding of participants was initially costly as the research team needed to travel long distances to meet face to face with the communities. Costs could be reduced in future iterations of the project if recruitment and onboarding activities are moved to the web-based environment.

As the intervention extended over a long period (32 weeks), not all participants were able to commit to taking part for the full length of time. Life events such as illness and changed personal, work, and social circumstances sometimes meant that participants needed to exit the study earlier than anticipated, resulting in less comprehensive data collection than was desired and planned for.

A strength and limitation of this project was the engagement of the *whole of rural community*; that is, multiple community stakeholders and community groups were involved. This was a strength as it meant that a variety of different users’ perspectives was evaluated and, practically, the implementation of the intervention did not have to be too tightly controlled. In a small rural community where the number of carers of people living with dementia is low, having an inclusive approach to recruitment increased the likelihood of recruiting a larger sample size. However, this approach was also limiting as it meant that information about caring and care support was broad. Thus, the care needs of individual carers may not have been met.

A limitation of the study was that randomization of communities was limited by the practical challenges of implementing the study. Owing to the nature of the intervention, the participants were not blinded to whether their community was in the control or intervention phase.

### Conclusions

This protocol provides an example of a study designed for real-world testing and the development of novel strategies intended to increase the support and information provided to informal carers of people living with dementia in small rural communities in Australia. This was a large and complex study addressing a health and care issue that will be of increasing significance to ageing societies such as Australia. This protocol illustrates some of the challenges and some examples of potential solutions for researchers who are engaging in complicated studies of intricate real-world situations.

## Appendix

Multimedia Appendix 1 Diagram of stepped-wedge control and implementation periods.

Multimedia Appendix 2 Screenshots of the Verily Connect app.

Multimedia Appendix 3 Participant information and consent form for carers.

Multimedia Appendix 4 Survey completed when carer participants first joined the study.

Multimedia Appendix 5 Survey completed by carer participants at data collection timepoints 3, 5, and 6.

Multimedia Appendix 6 Focus group question guide for volunteer participants.

Multimedia Appendix 7 Interview question guide for feedback from all participants at follow-up.

Multimedia Appendix 8 Interview question guide for staff participants based on the Consolidated Framework of Implementation Research.

Multimedia Appendix 9 CONSORT-eHEALTH checklist (V 1.6.1).

## Abbreviations

CFIR: Consolidated Framework of Implementation Research
ICT: information and communication technology
MOS-SSS: Medical Outcomes Study-Social Support Survey
ZBI: Zarit Burden Interview
