# Supplementary figures and images for: Web-Based Technologies to Support Carers of People Living With Dementia: Protocol for a Mixed Methods Stepped-Wedge Cluster Randomized Controlled Trial
Source: JMIR Res Protoc. 2022 May 19;11(5):e33023. doi: 10.2196/33023 (PMC9164093; doi:10.2196/33023)

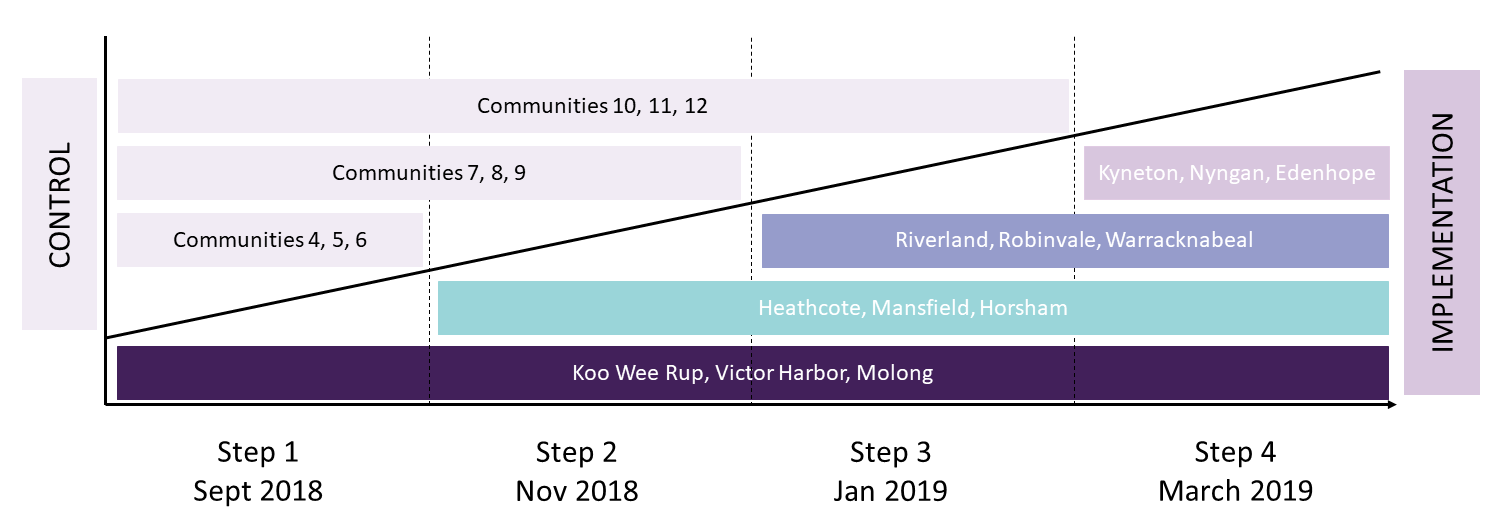

Supplement: Multimedia Appendix 1 [file resprot_v11i5e33023_app1.png]
