# Supplementary material for: Web-Based Technologies to Support Carers of People Living With Dementia: Protocol for a Mixed Methods Stepped-Wedge Cluster Randomized Controlled Trial
Source: JMIR Res Protoc. 2022 May 19;11(5):e33023. doi: 10.2196/33023 (PMC9164093; doi:10.2196/33023)
Supplement: Multimedia Appendix 2 [file resprot_v11i5e33023_app2.pdf]

Verily Connect Website/app

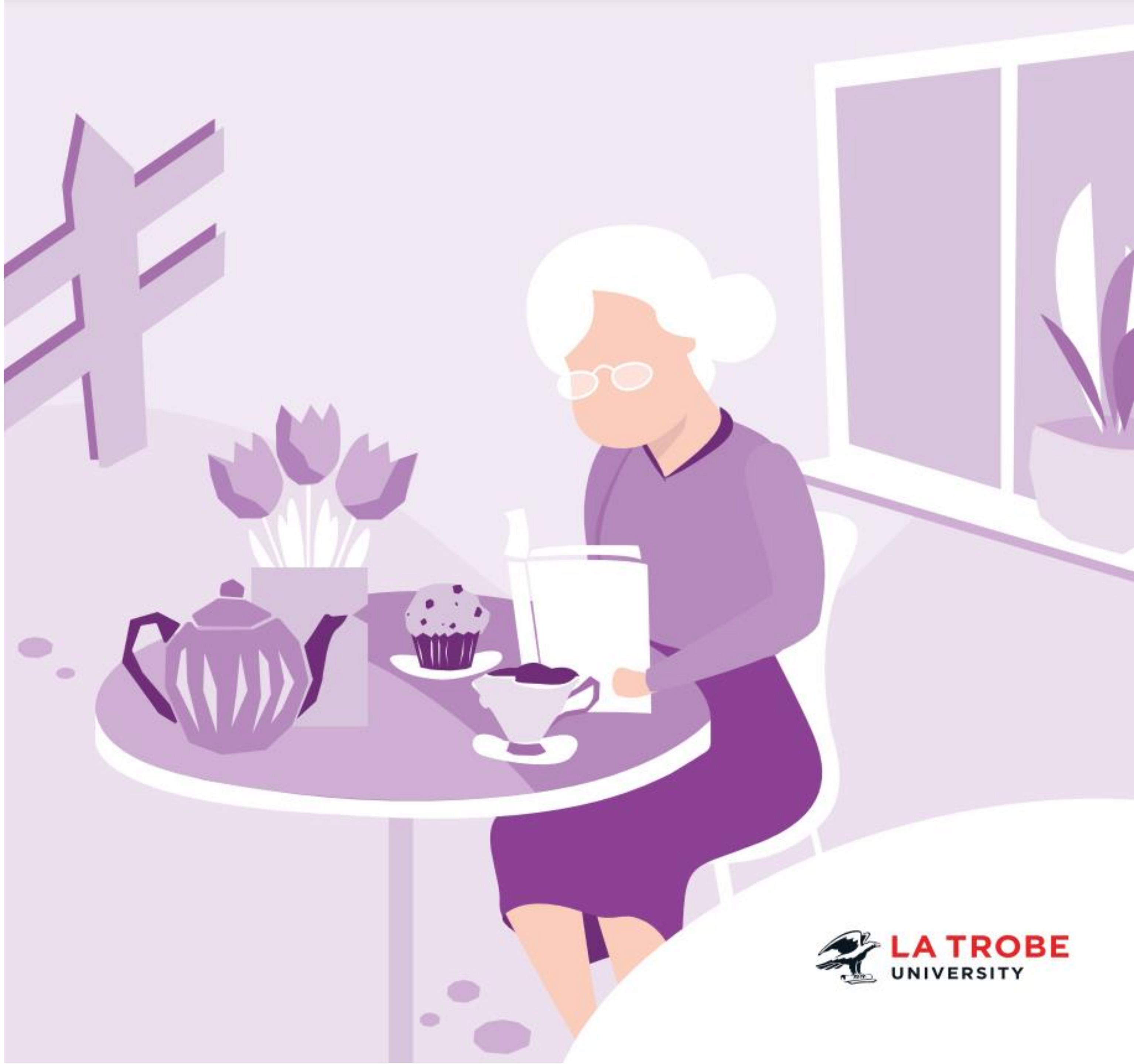

LA TROBE UNIVERSITY

Landing page

verilyconnect

Welcome to Verily Connect

VerilyConnect is an online meeting place for carers to support each other in a dementia-friendly community.

Resources | Disclaimer

About

GET IT ON Google Play | Download on the App Store

LA TROBE UNIVERSITY

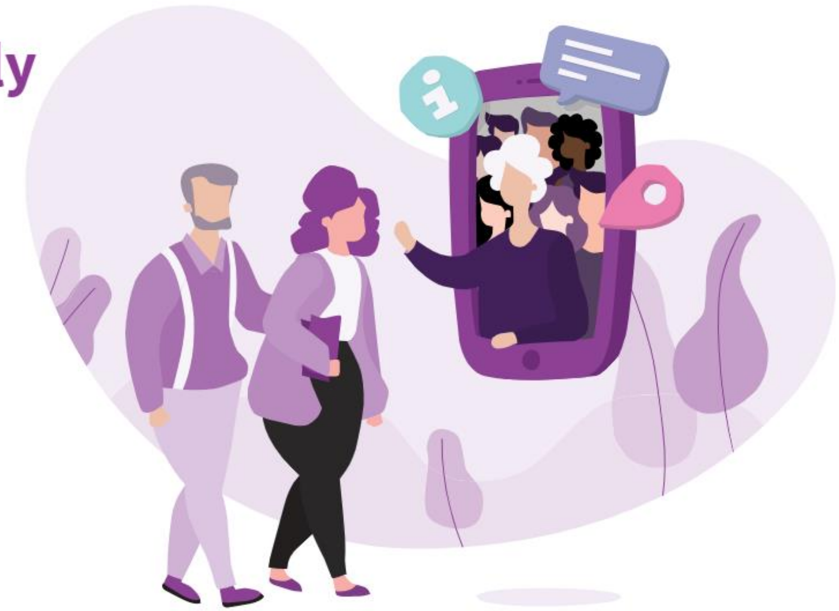

Menu page

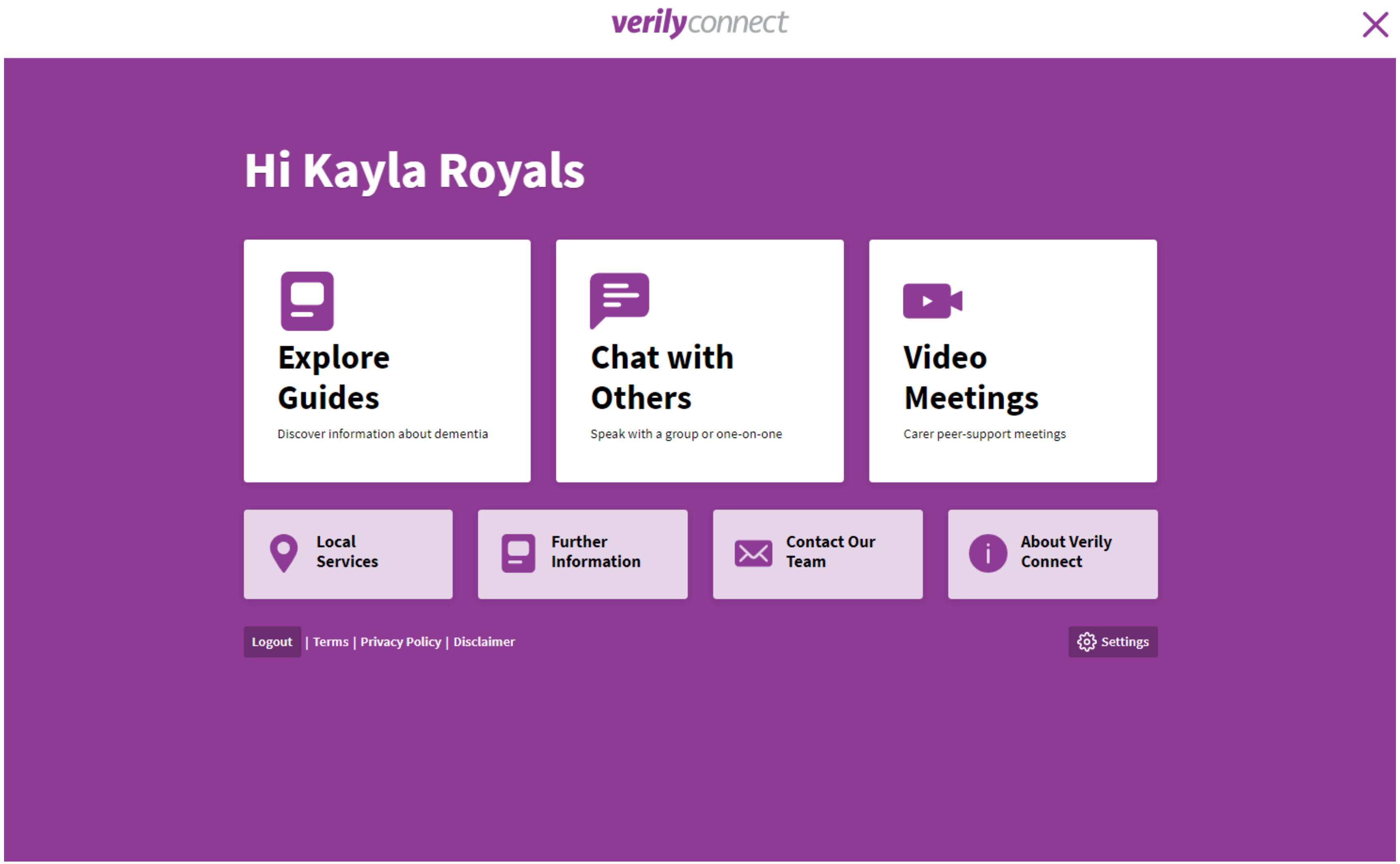
verilyconnect
Hi Kayla Royals
Explore Guides
Discover information about dementia
Chat with Others
Speak with a group or one-on-one
Video Meetings
Carer peer-support meetings
Local Services
Further Information
Contact Our Team
About Verily Connect
Logout
| Terms | Privacy Policy | Disclaimer
Settings

Guides

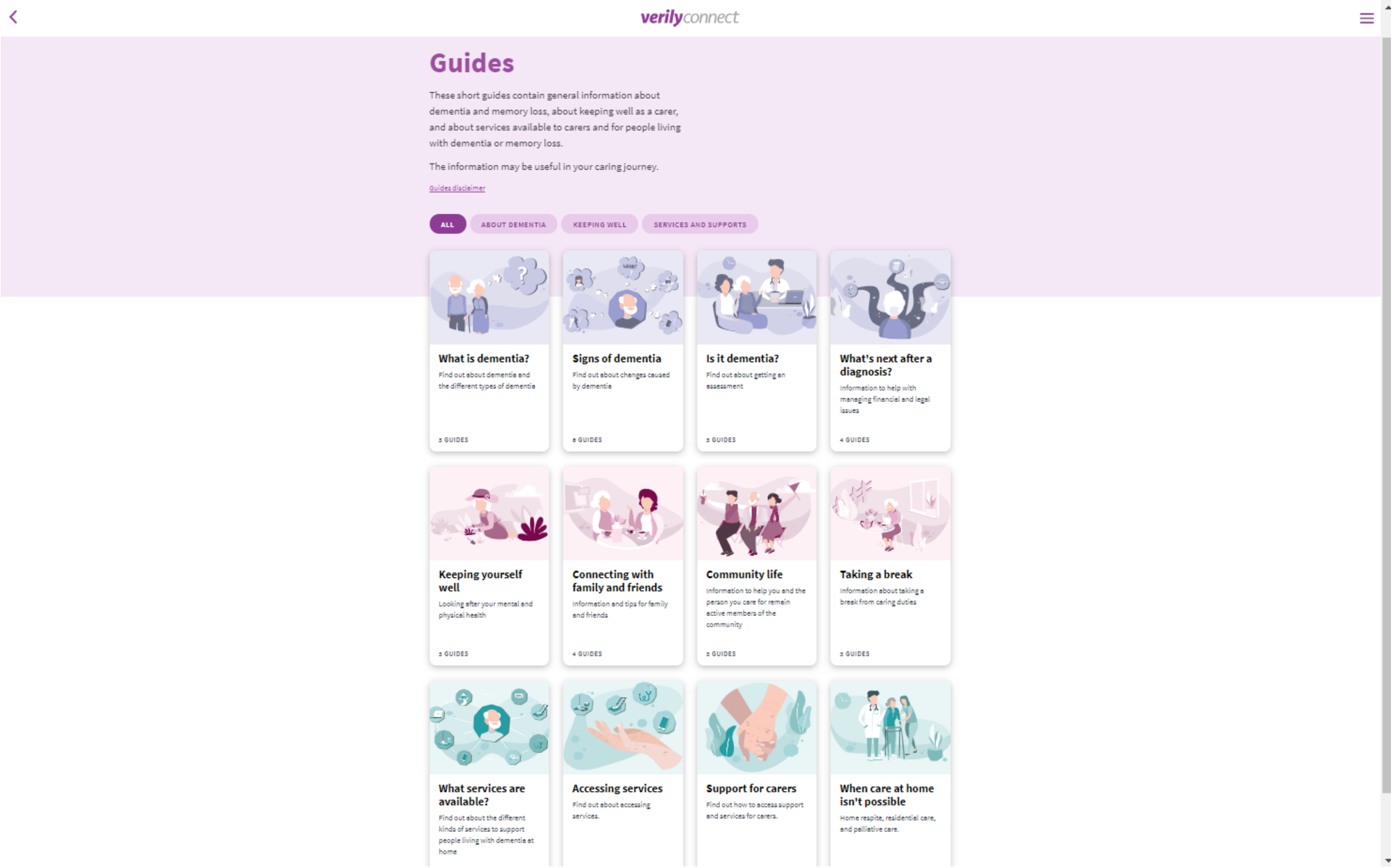

verilyconnect
Guides
These short guides contain general information about dementia and memory loss, about keeping well as a carer, and about services available to carers and for people living with dementia or memory loss.
The information may be useful in your caring journey.
Guides disclaimer
ALL
ABOUT DEMENTIA
KEEPING WELL
SERVICES AND SUPPORTS
What is dementia?
Find out about dementia and the different types of dementia
Signs of dementia
Find out about changes caused by dementia
Is it dementia?
Find out about getting an assessment
What's next after a diagnosis?
Information to help with managing financial and legal issues
4 GUIDES
Keeping yourself well
Looking after your mental and physical health
Connecting with family and friends
Information and tips for family and friends
4 GUIDES
Community life
Information to help you and the person you care for remain active members of the community
Taking a break
Information about taking a break from caring duties
What services are available?
Find out about the different kinds of services to support people living with dementia at home
Accessing services
Find out about accessing services.
Support for carers
Find out how to access support and services for carers.
When care at home isn't possible
Home respite, residential care, and palliative care.

Guides – What’s next after a diagnosis?

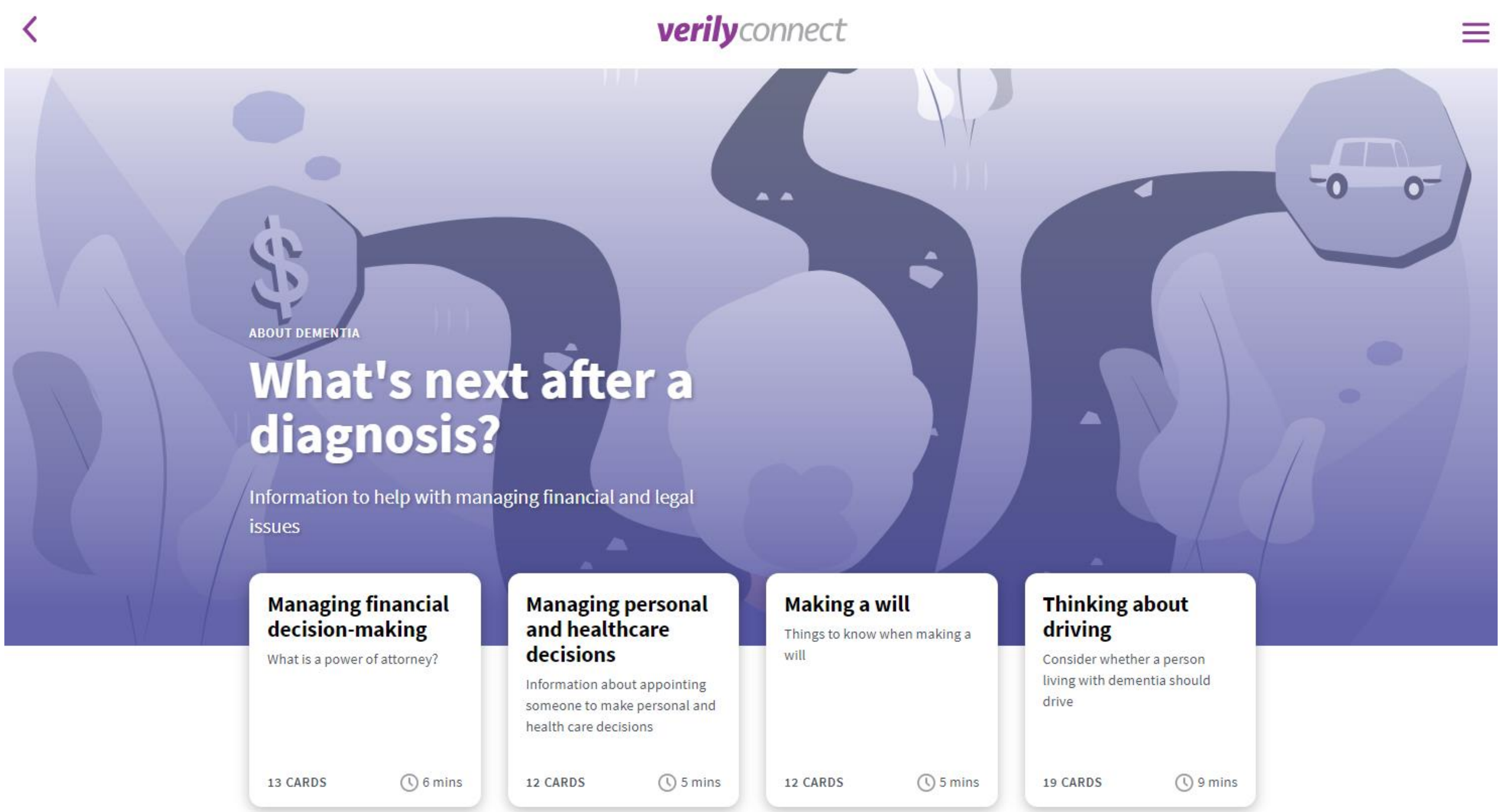

Guide – Thinking about driving

verilyconnect

GUIDES > WHAT'S NEXT AFTER A DIAGNOSIS?

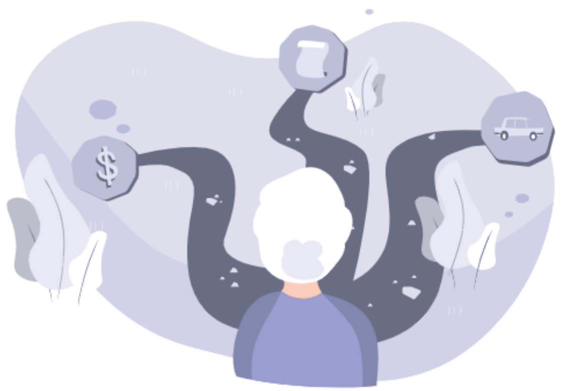

Thinking about driving

Consider whether a person living with dementia should drive

CARD 1/19

Guide – Thinking about driving

verilyconnect

GUIDES > WHAT'S NEXT AFTER A DIAGNOSIS?

All drivers are required by law to inform the local licensing authority (e.g. VicRoads, Service NSW, Service SA) about any medical conditions that affect driving, including dementia.

CARD 5/19

Chat (all topics)

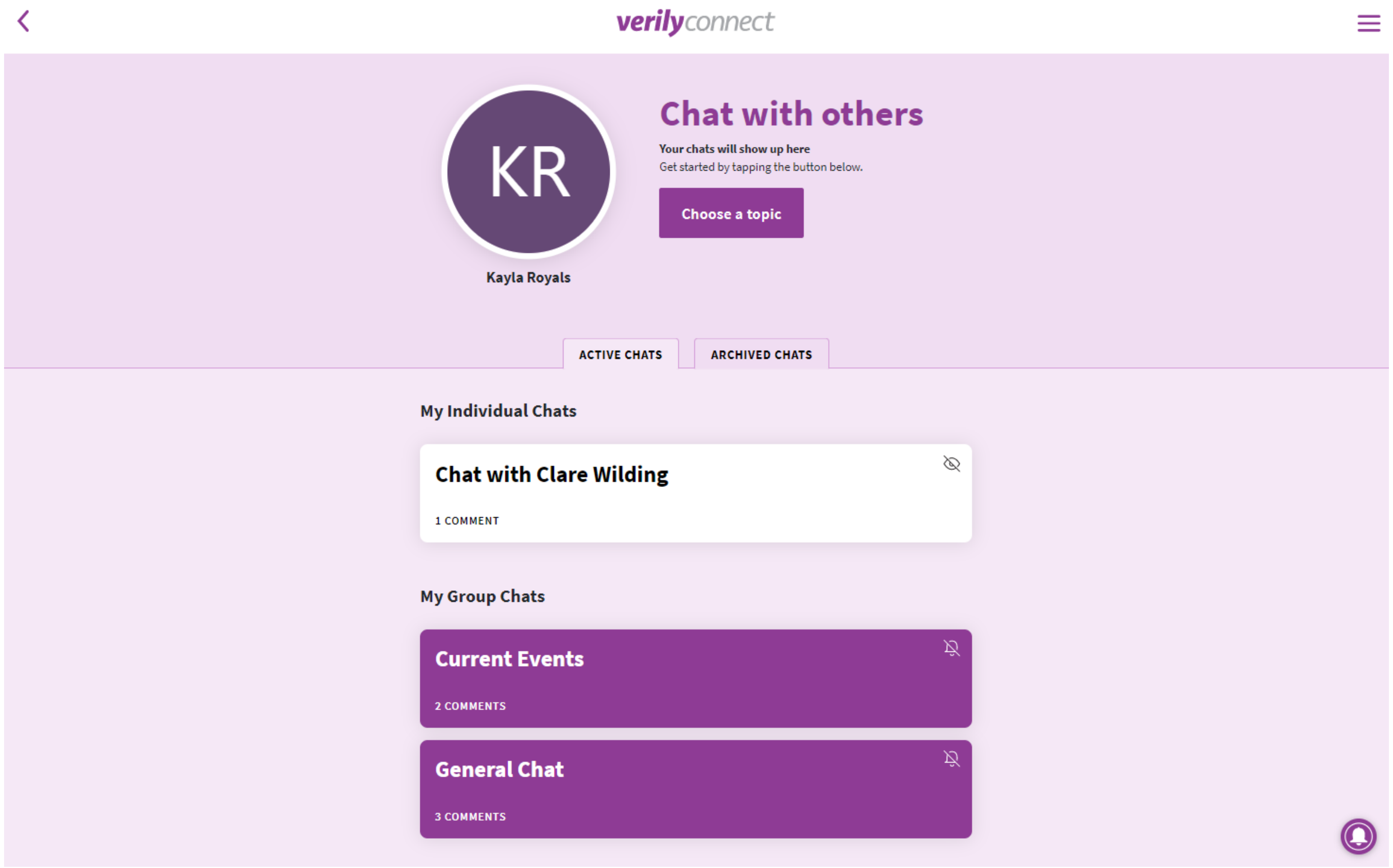

Chat – Current Events group chat

verilyconnect

GROUP CHAT

Current Events

CW Clare Wilding | Feb 12th, 2019 at 11:53am

Hello, you might be interested in learning more about dementia. The Wicking Dementia Research and Education Centre at the University of Tasmania offers 2 fantastic massive open online courses (MOOCs) that are free short online courses. They consist of a series of videos and other activities. The technology is very easy to use (if you need help, we can always put you in touch with a volunteer in your local community who can provide face-to-face help).
The Understanding Dementia MOOC is about to start again. It is a really excellent course. You can learn more and enrol for the course at https://mooc.utas.edu.au/courses/understanding-dementia-2019-02

CHAT PRIVATELY

CW Clare Wilding | Sep 3rd, 2018 at 9:25am

Hello and Welcome to "current events". This is a place to chat about upcoming events - in your community, your state, or even a National event. For example, September 2018 is National Dementia Awareness month. You can visit the Dementia Australia calendar to find out more about what's happening: https://www.dementia.org.au/calendar

CHAT PRIVATELY

Write a comment...

Submit

Video meetings page

verilyconnect

Video meetings

Video-conference meetings are a way for carers to connect with other carers and to participate in meetings with service providers.

In these meetings you can swap stories and information, ask and answer questions, and give and receive support from other carers.

Verily Connect video-conferences are similar to carer support groups that you may already belong to – it's just that we meet online rather than in a physical location.

Join a meeting – meet and make new friends – share your experiences with others who are in a similar situation to you.

Zoom

We are using video-conferencing software called Zoom for the meetings.

Zoom is free to download and use. The only cost is for the data that is used during the meeting.

If you are worried about data costs, you may like to visit a Verily Connect technology support hub (if available in your town) to find out about any free or subsidised options for connecting to the meetings.

To download and connect to Zoom on a computer, please visit: https://support.zoom.us/hc/en-us/articles/201362033-Getting-Started-on-PC-and-Mac

To use Zoom on a mobile device, go to the App Store or Play Store and download the app called "Zoom"

Upcoming meetings

WHEN

There are currently no events scheduled. Stay tuned for more events soon.

Local services (all)

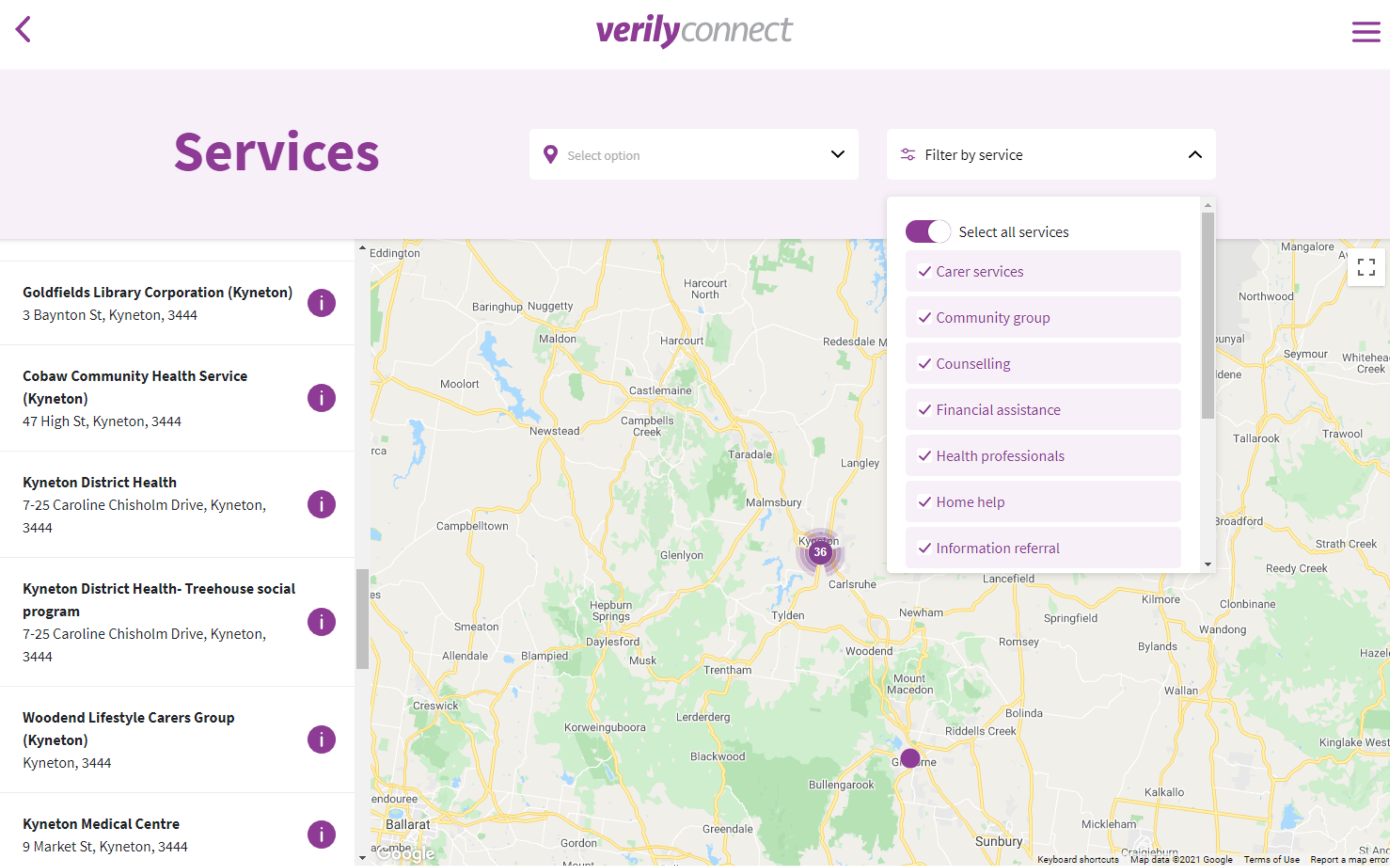

Local services (Kyneton library)

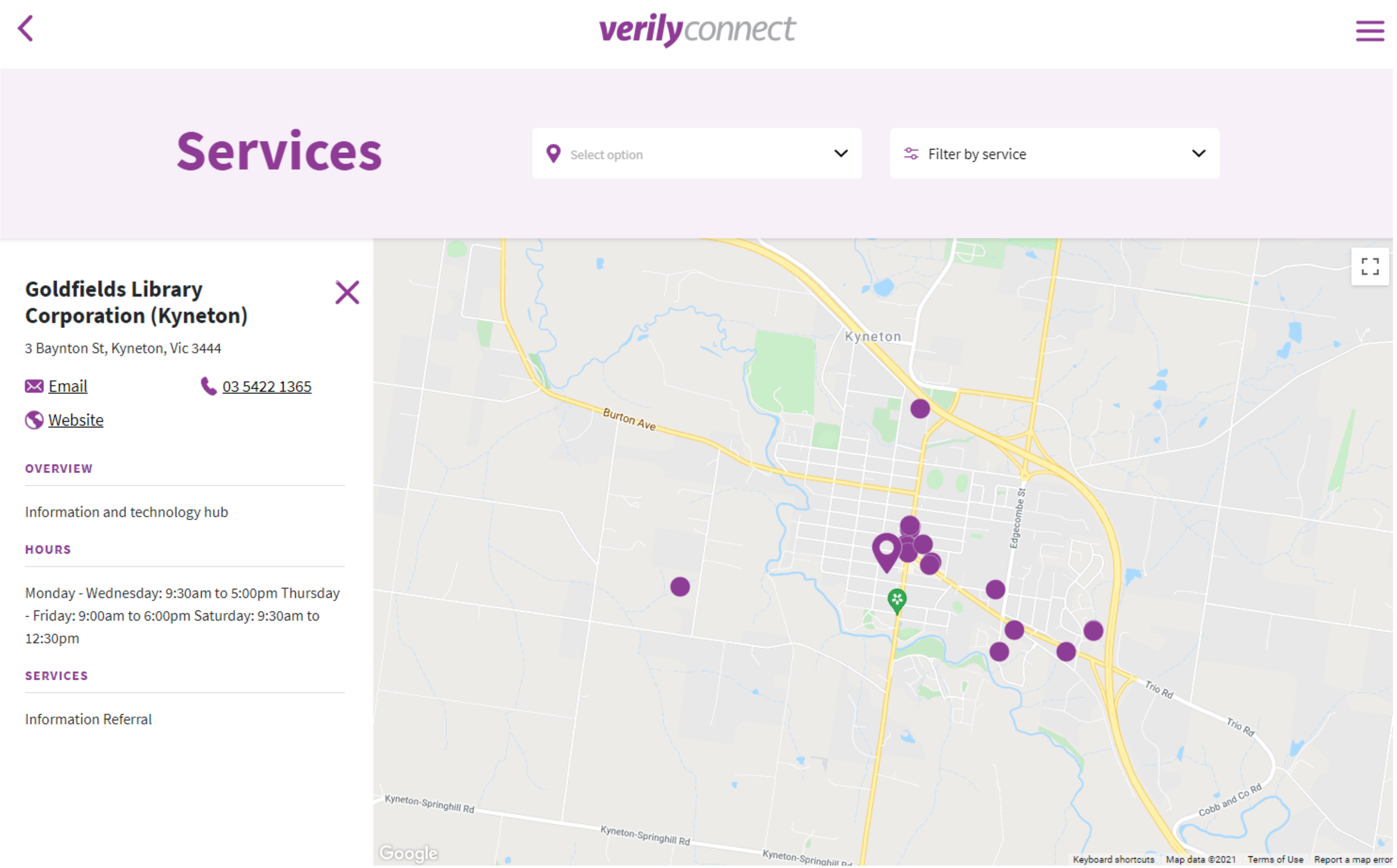

Further information (topic search)

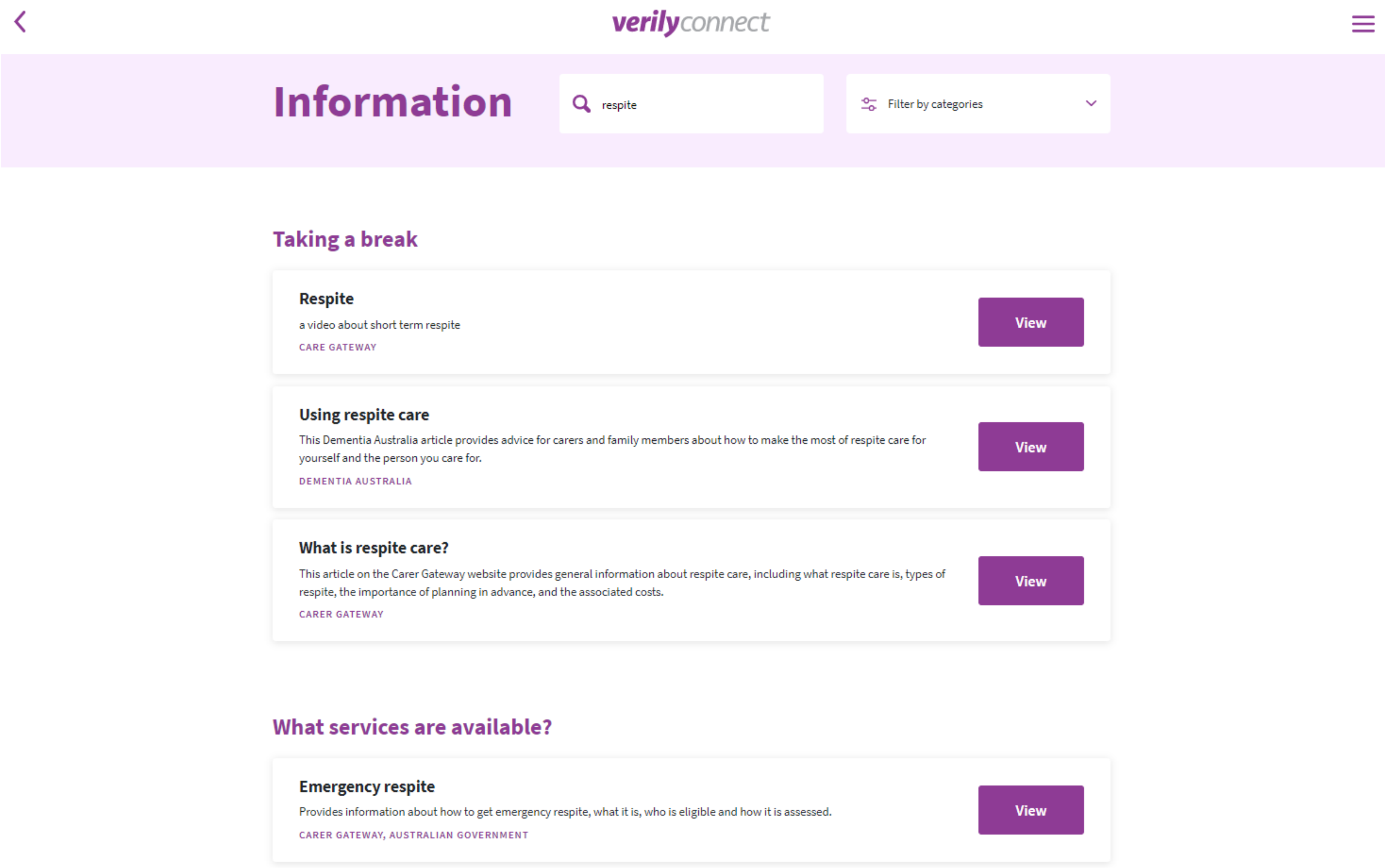

Further information (topic selection)

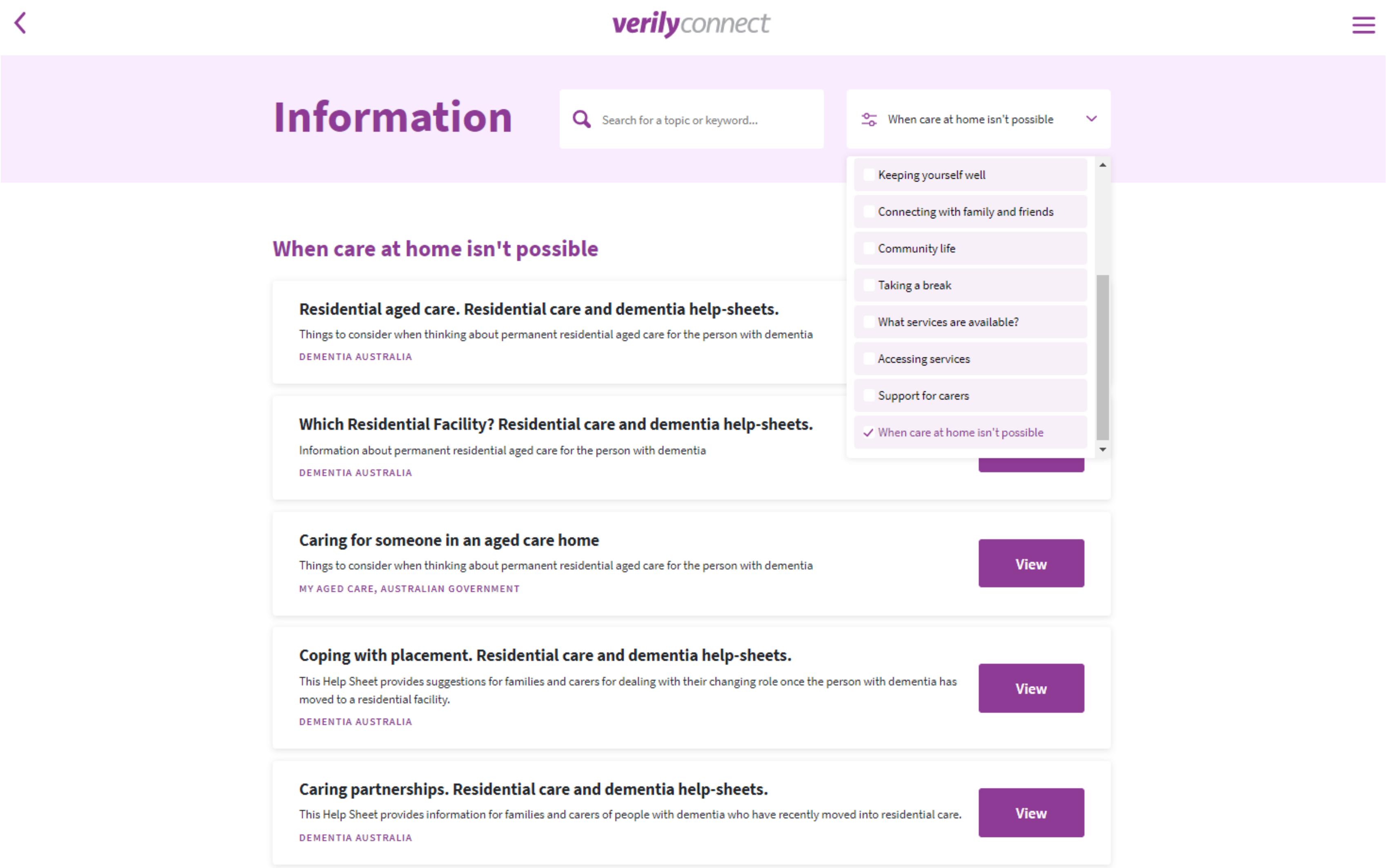

Contact us page

verilyconnect

Verily Connect: An Australian Government Initiative

Verily Connect is a project to trial some innovative solutions for increasing support for rural carers of people living with dementia and/or memory problems. We use online technologies to connect carers to each other and to create virtual dementia friendly rural communities – that is, an online dementia-friendly community.

Verily Connect consists of three main strategies:

1. Real-world Technology Learning Centres manned by trained volunteers who can assist carers and community members to learn to use Verily Connect technologies
2. The Verily Connect website and mobile application that provides information for carers and helps them connect with each other
3. Peer support groups for carers that meet via video-conference

A total of 12 rural communities across Victoria, South Australia, and New South Wales are participating in the project. The project runs from July 2017 – December 2019.

La Trobe University is leading the project in collaboration with Swinburne University, Flinders University, University of Newcastle, and University of Saskatchewan (Canada). The project is funded by the Australian Government Department of Health under the Dementia and Aged Care Services (DACS) funding round.

The ethical aspects of this study have been approved by the Human Research Ethics Committee of Melbourne Health. This project will be carried out according to the *National Statement on Ethical Conduct in Human Research (2007)*. This statement has been developed to protect the interests of people who agree to

Get in touch

(02) 6024 9718

verilyconnect@latrobe.edu.au

Please fill out the enquiry form below to contact us.
We'll get in touch with you as soon as possible.

Name

Phone number

Email

Message

Submit

LA TROBE UNIVERSITY

About page

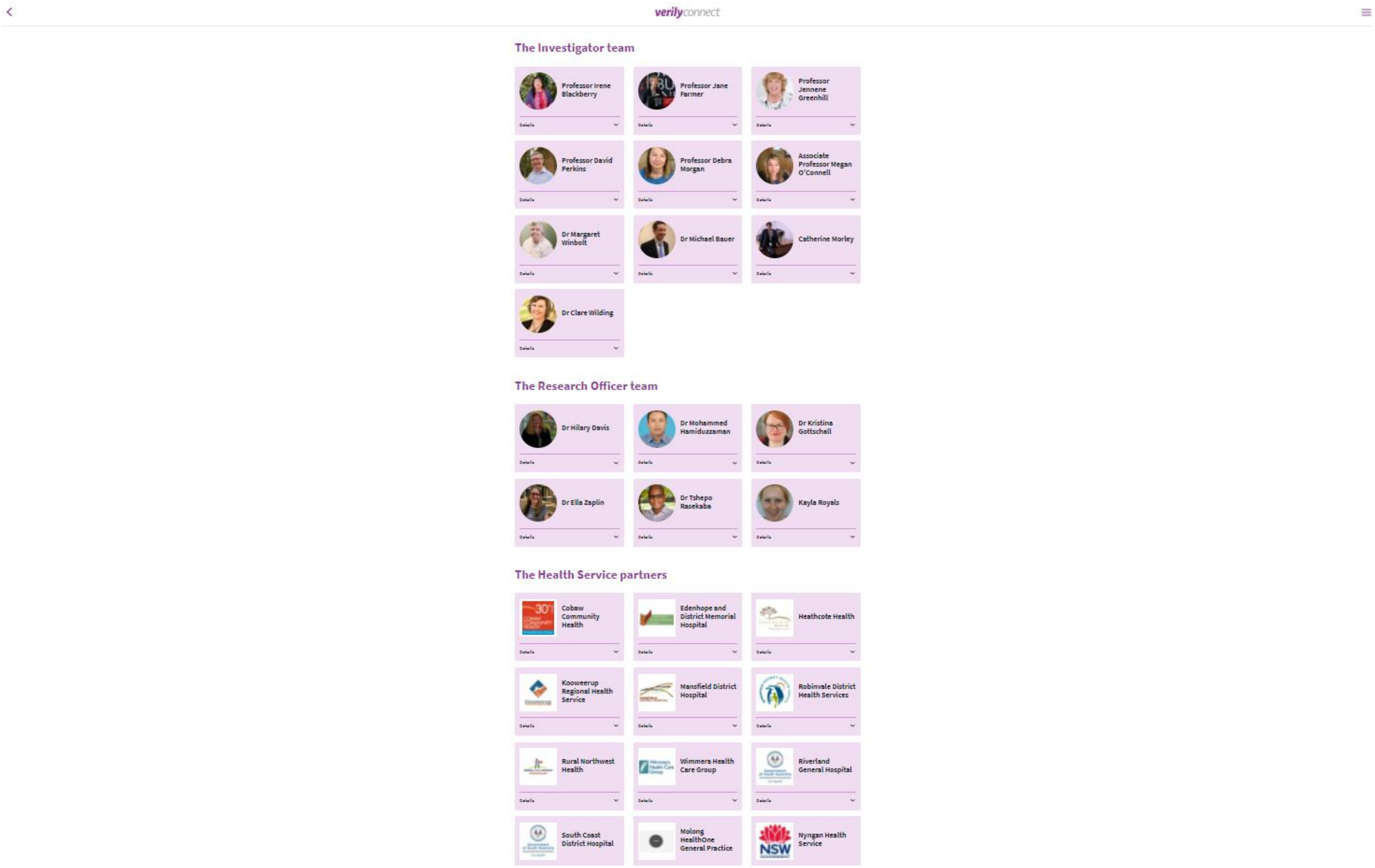
verilyconnect
The Investigator team
Professor Irene Blackberry
Professor Jane Farmer
Professor Jennene Greenhill
Professor David Perkins
Professor Debra Morgan
Associate Professor Megan O'Connell
Dr Margaret Winbolt
Dr Michael Bauer
Catherine Morley
Dr Clare Wilding
The Research Officer team
Dr Hilary Davis
Dr Mohammed Hamiduzzaman
Dr Kristina Gottschall
Dr Ella Zaplin
Dr Tshepo Rasekaba
Kayla Royals
The Health Service partners
Cobaw Community Health
Edenhope and District Memorial Hospital
Heathcote Health
Kooweerup Regional Health Service
Mansfield District Hospital
Robinvale District Health Services
Rural Northwest Health
Wimmera Health Care Group
Riverland General Hospital
South Coast District Hospital
Molong HealthOne General Practice
Nyngan Health Service
NSW
