# Supplementary material for: Web-Based Technologies to Support Carers of People Living With Dementia: Protocol for a Mixed Methods Stepped-Wedge Cluster Randomized Controlled Trial
Source: JMIR Res Protoc. 2022 May 19;11(5):e33023. doi: 10.2196/33023 (PMC9164093; doi:10.2196/33023)
Supplement: Multimedia Appendix 3 [file resprot_v11i5e33023_app3.pdf]

LA TROBE UNIVERSITY

John Richards Centre
for Rural Ageing Research
College of Science, Health and Engineering
Albury-Wodonga Campus

PO Box 821, Wodonga
Victoria 3689 Australia
T +61 2 6024 9730
F +61 2 6024 9797
E jrc@latrobe.edu.au
www.latrobe.edu.au/jrc

Participant Information Sheet for carers

Health/Social Science Research - *Adult providing own consent*

**Title**	Virtual Dementia Friendly Rural Communities (VERILY) project
**Short Title**	VERILY
**Project Sponsor**	Australian Government Dementia and Aged Care Services Fund: Research and Innovation Grant
**Coordinating Principle Investigator/Principle Investigators**	Professor Irene Blackberry<br>Professor Jane Farmer<br>Professor Jennene Greenhill<br>Professor David Perkins<br>Professor Debra Morgan<br>Associate Professor Megan O'Connell<br>Ms Catherine Morley<br>Dr Margaret Winbolt<br>Dr Michael Bauer<br>Dr Clare Wilding
**Research Officers**	Dr Hilary Davis<br>Dr Tshepo Rasekaba<br>Miss Ainsley Robinson<br>Dr Kristina Gottschall<br>Mr Mohammad Hamiduzzaman<br>Ms Kayla Royals

Part 1 What does my participation involve?

1 Introduction

You are invited to take part in this research project, which is called Virtual Dementia Friendly Rural Communities (VERILY) project. You have been invited because you care for someone with dementia or cognitive impairment. Your contact details were obtained from the expression of interest postcard you returned.

This Participant Information Sheet tells you about the research project. It explains the processes involved with taking part. Knowing what is involved will help you decide if you want to take part in the research.

Please read this information carefully. Ask questions about anything that you don't understand or want to know more about. Before deciding whether or not to take part, you might want to talk about it with a relative, friend or local health worker.

Participation in this research is voluntary. If you don't wish to take part, you don't have to.

If you decide you want to take part in the research project, you will be asked to sign the consent section. By signing it you are telling us that you:

- Understand what you have read
- Consent to take part in the research project
- Consent to be involved in the research described
- Consent to the use of your personal and health information as described.

You will be given a copy of this Participant Information and Consent Form to keep.

2 What is the purpose of this research?

The VERILY project is about supporting people who are caring for someone with dementia in a rural setting and helping rural communities to better understand and support people with dementia and their family and friends.

This research trials and evaluates innovative technology and skills-building to increase the capacity of rural communities to provide support for and care of local people who have dementia, and their carers, family and friends. We need your involvement to help us develop, co-design, and test these technologies.

Specifically, there are 3 strategies being trialled during VERILY:

1. A "technology learning hub" in your community that is staffed by volunteers who have been trained to help carers to learn how to use online technology
2. A website and mobile application (app) (called "Verily Connect") that provides information about services for people with dementia and that also helps connect carers and service providers with each other
3. Carer peer support groups that meet by video-conference

At the conclusion of this project, we plan to develop a toolkit for other rural Australian communities to implement their own virtual dementia-friendly communities.

This research is funded by an Australian Government Dementia and Aged Care Services Fund: Research and Innovation Grant.

3 What does participation in this research involve?

You will need to sign a Consent Form at the beginning of your involvement.

We are seeking the following people as participants in the study:

- You are caring for a person with dementia or cognitive impairment
- You live in one of the 12 rural communities participating in the VERILY project
- You are willing to try using the VERILY app on a smartphone or tablet with internet access or to use the website on a computer with internet access
- You are willing to participate in at least one peer support group via video-conference on a computer or mobile device with internet access and video-conferencing functionality

There are 12 rural communities participating in the VERILY project. These communities are: in Victoria - Heathcote, Horsham, Kyneton, Robinvale, Mansfield, Koo Wee Rup, Edenhope and Warracknabeal; in New South Wales – Molong and Nyngan; and in South Australia – Riverland and Victor Harbour.

You will be asked to complete a questionnaire 5 times during the study (in August, October and November 2018 and January and March in 2019). You can complete the questionnaire online or by post. A researcher will contact you each time a questionnaire is due to be completed. Three of the questionnaires will take approximately 30 minutes to complete on each occasion, and two

of the questionnaires will take approximately 15 minutes to complete on each occasion, which is a total of approximately 2 hours.

From when VERILY activities begin in your community, we ask you to log on to the Verily Connect website and/or mobile application at least 4 times. You can decide how long or how little time you spend using the website/app on each occasion, but we suggest you might find it useful to spend about 30 minutes each time. If you want, you can contact the technology learning hub in your community to receive assistance in learning how to use the website/app. Local community volunteers will be available to assist you in using these technologies. And, each hub will each have an iPad and Samsung phone that you can use to log on to the app.

You will be invited to participate in online, video-conferenced peer support meetings that help you to connect with other rural carers of people with dementia. Times that the groups are available will be published in a special section of the Verily Connect website/app. Groups will be available for you to attend every two months. Each group will last about an hour. We ask that you attend at least one group. You can use your own computer or mobile device to join the video-conference group or you can contact your local technology learning hub to find out about other options available in your community. Local community volunteers will be available to assist you to join the group meetings.

At least every 8 weeks, a researcher will contact you by telephone and ask if you have any feedback, suggestions, ideas, or problems about your participation in the VERILY project. The researcher will make notes about the phone call that will be collected as data. In these phone calls, you are free to decide for how long you talk and what you say to the researcher.

We will also be collecting information about how VERILY is used, including collecting digital information about your use of the website/app. You do not need provide this information, it will be collected automatically by the website/app software. Any public comments made using VERILY technology will also be collected as part of the data for the study.

This research project has been designed to make sure the researchers interpret the results in a fair and appropriate way and avoids researchers or participants jumping to conclusions.

Access to the Verily Connect website, mobile app, and video-conferenced support groups is free; however, if you use your own computer or mobile device you will be responsible for the related data download costs with your internet provider. In appreciation of your participation in VERILY and research activities you will be given gift cards as reimbursement for your time. You will receive a gift card to the value of $100 in June 2019 and another to the value of $50 in September 2019.

We are extending the VERILY project beyond the original completion date of April 2019, and there are two additional research activities:

In April – June 2019, a researcher will contact you and invite you to participate in a one-to-one interview that will happen either face-to-face or by telephone, in your home or in a private room at your nearest health service, as mutually agreed by you and the researcher. These interviews will take approximately 20 – 45 minutes, depending how much you would like to say. The interviews will ask your feedback about the VERILY activities you have participated in and about your needs as a carer. You decide how long you talk and what you say to the researcher. The interview will be audio-recorded.

In August – September 2019, a researcher will contact you and invite you to complete a questionnaire that will take approximately 30 minutes to complete and you will be invited to complete a short phone interview that will take approximately 10 – 20 minutes. The interview asks your feedback about your use of VERILY activities in the months from April – August 2019. The interview will be audio-recorded.

4 Other relevant information about the research project

In each of the 12 participating communities, we will be seeking feedback from 15 carers, 5 volunteers, and 3 service provider staff. Each group of participants will have a different role in the study.

The project involves collaboration between researchers from La Trobe University, Swinburne University, University of Newcastle, Flinders University, University of Saskatchewan, and Wimmera Health Care Group.

This project builds on the work of a pilot study completed in 2017 that was called the Service Navigation and Networking for Rural Communities (SENDER) app. In that study a prototype mobile app was developed. The VERILY study takes the learning from the SENDER app as a starting point for creating a more comprehensive and integrated way of using online technologies to support rural carers of people living with dementia.

5 Do I have to take part in this research project?

Your participation in this study is voluntary. There are no disadvantages, penalties or adverse consequences for not participating. You have the right to withdraw from participation in this project at any time. However, we cannot remove any data that you have provided up until you decide to withdraw. We will be analysing and using the data during the project to develop and improve the project technologies and actions.

Your decision whether to take part or not to take part, or to take part and then withdraw, will not affect your routine care, your relationship with professional staff or your relationship with La Trobe University.

6 What are the possible benefits of taking part?

We cannot guarantee or promise that you will receive any benefits from this research; however, possible benefits may include learning more about the services that are available in your local community. You may also find the experience of using the website or app and participating in the peer support groups to be helpful. You may feel less isolated and more supported.

7 What are the possible risks and disadvantages of taking part?

Participation in VERILY activities is likely to take a minimum of 8 hours, and may take more if you choose to spend more time. If you decide to participate, you would be enrolled in the study until April 2019, and researchers will be in contact with you approximately every 2 months to ask you to complete questionnaires and to seek your feedback about the project. If you decide to participate in the extended VERILY study, you would be enrolled in the study until September 2019; during this period you will be contacted by researchers to invite you to participate in the interview in April-May that lasts 20-45 minutes and the questionnaire and interview in August-September that takes approximately 40-50 minutes.

Although VERILY activities are designed to assist carers of people with dementia, you may find that they are not helpful to you in your situation.

We do not anticipate that participation in this study will be distressing; however, you will be asked questions about how you feel about caring for a person with dementia, the support you receive from others, and how connected you feel to others – sometimes considering these questions can raise unexpected reactions in people. There are no right or wrong answers and you do not have to answer any questions that you do not wish to. You may feel that some of the questions we ask are stressful or upsetting. If you do not wish to answer a question, you may skip it and go to the next question, or you may stop immediately. If you need support you may call the National Dementia Helpline: 1800 100 500 or Carers Australia: 1800 242 636.

During the peer group discussions we will ask all participants to respect the privacy and confidentiality of the other people in the group. However, you may experience embarrassment if one of the group members were to repeat things said in a confidential group meeting.

8 What if I withdraw from this research project?

You may withdraw from this project at any time. If you decide to withdraw from the project, please notify a member of the research team. If you do withdraw, you will be asked to complete and sign a 'Withdrawal of Consent' form; this will be provided to you by the research team.

If you decide to leave the research project, the researchers will not collect additional personal information from you, although personal information already collected will be retained to ensure that the results of the research project can be measured properly and to comply with law. We cannot remove any data that you have provided up until you decide to withdraw. Data collected up to the time you withdraw will form part of the research project results.

9 Could this research project be stopped unexpectedly?

It is unlikely that this research project will stop unexpectedly although this could occur if the funding for the project was unexpectedly terminated.

10 What happens when the research project ends?

If you wish, you will be provided with a one page summary of the findings. You may also request the results of your individual questionnaires. These results will be emailed or posted to you in July 2019.

In August-September 2019, findings from the study will also be presented in public community forums in each of the 12 participating communities.

Part 2 How is the research project being conducted?

11 What will happen to information about me?

By signing the consent form, you consent to the research team collecting and using health and personal information about you for the research project. Any information obtained in connection with this research project that can identify you will remain confidential. Information that is collected as part of this study will be coded, which means that it will be re-identifiable. Data will be kept in digital format on the La Trobe University server. Data will only be accessible to the VERILY research team.

The data will be stored for a period of 15 years and then destroyed, with the exception of de-identified survey data (this is data that is not individually identifiable), which will remain available in the La Trobe University Library Research Data Portal. The data for this project will not be used for any extended research or unspecified future research. Data collected from South Australia and New South Wales will be collected and stored in Victoria, as La Trobe University is an organisation that is based in Victoria.

The information collected during this project will be presented as a final report, academic papers, project communications (newsletters, blog reports), and conference presentations. Any reports that arise from this study will not identify individuals, except with your express permission. Your identity will be protected by using pseudonyms and reporting aggregated data.

In accordance with Australian privacy law, you have the right to request access to the information about you that is collected and stored by the research team. You also have the right to request that any information with which you disagree be corrected. Please contact Dr Clare

Wilding, John Richards Centre, La Trobe University, PO Box 821, Wodonga, Victoria, 3689 or M:0428 557 584 or P:02 6024 9651 or E:c.wilding@latrobe.edu.au if you would like to access your information. Upon request, Dr Wilding can provide you with a copy of the transcript of your interviews.

Any information obtained for the purpose of this research project that can identify you will be treated as confidential and securely stored. It will be disclosed only with your permission, or as required by law.

12 Complaints and compensation

What if I have questions or need further information?
If you have any questions, please contact Dr Clare Wilding, John Richards Centre, La Trobe University, PO Box 821, Wodonga, Victoria, 3689 or M:0428 557 584 or P:02 6024 9651 or E:c.wilding@latrobe.edu.au

If you have any complaints or concerns about your participation in the study that the researcher has not been able to answer to your satisfaction, you may contact:

Reviewing HREC name:	Melbourne Health HREC
HREC Executive Officer:	Manager HREC
Telephone:	(03) 9342 8530
Email:	Research@mh.org.au

13 Who is organising and funding the research?

La Trobe University is leading this research project in partnership with Swinburne University, Flinders University, University of Newcastle, and University of Saskatchewan (Canada). An Australian Government Dementia and Aged Care Services Fund: Research and Innovation Grant is funding the study.

14 Who has reviewed the research project?

All research in Australia involving humans is reviewed by an independent group of people called a Human Research Ethics Committee (HREC). The ethical aspects of this research project have been approved by the HREC of Melbourne Health. This project will be carried out according to the *National Statement on Ethical Conduct in Human Research (2007)*. This statement has been developed to protect the interests of people who agree to participate in human research studies.

15 Further information and who to contact

The person you may need to contact will depend on the nature of your query. If you want any further information concerning this project or if you have any problems which may be related to your involvement in the project, you can contact Dr Clare Wilding, John Richards Centre, La Trobe University, PO Box 821, Wodonga, Victoria, 3689 or M:0428 557 584 or P:02 6024 9651 or E:c.wilding@latrobe.edu.au

LA TROBE UNIVERSITY

John Richards Centre
for Rural Ageing Research
College of Science, Health and Engineering
Albury-Wodonga Campus

PO Box 821, Wodonga
Victoria 3689 Australia
T +61 2 6024 9730
F +61 2 6024 9797
E jrc@latrobe.edu.au
www.latrobe.edu.au/jrc

Consent Form - *Adult providing own consent*

**Title**	Virtual Dementia Friendly Rural Communities (VERILY) project
**Short Title**	VERILY
**Project Sponsor**	Australian Government Dementia and Aged Care Services Fund: Research and Innovation Grant
**Coordinating Principle Investigator/Principle Investigators**	Professor Irene Blackberry<br>Professor Jane Farmer<br>Professor Jennene Greenhill<br>Professor David Perkins<br>Professor Debra Morgan<br>Associate Professor Megan O'Connell<br>Ms Catherine Morley<br>Dr Margaret Winbolt<br>Dr Michael Bauer<br>Dr Clare Wilding
**Research Officers**	Dr Hilary Davis<br>Dr Tshepo Rasekaba<br>Miss Ainsley Robinson<br>Dr Kristina Gottschall<br>Mr Mohammad Hamiduzzaman<br>Ms Kayla Royals

Declaration by Participant

I have read the Participant Information Sheet or someone has read it to me in a language that I understand.

I understand the purposes, procedures and risks of the research described in the project.

I have had an opportunity to ask questions and I am satisfied with the answers I have received.

I freely agree to participate in this research project as described and understand that I am free to withdraw at any time during the project without affecting my future care.

I understand that I will be given a signed copy of this document to keep.

Name of Participant (please print) ______________________________

Signature ______________________ Date ______________________

Declaration by Researcher

I have given a verbal explanation of the research project, its procedures and risks and I believe that the participant has understood that explanation.

Name of Researcher (please print) ______________________________

Signature ______________________ Date ______________________

John Richards Centre
for Rural Ageing Research
College of Science, Health and Engineering
Albury-Wodonga Campus

PO Box 821, Wodonga
Victoria 3689 Australia
T +61 2 6024 9730
F +61 2 6024 9797
E jrc@latrobe.edu.au
www.latrobe.edu.au/jrc

Form for Withdrawal of Participation - *Adult providing own consent*

**Title**	Virtual Dementia Friendly Rural Communities (VERILY) project
**Short Title**	VERILY
**Project Sponsor**	Australian Government Dementia and Aged Care Services Fund: Research and Innovation Grant
**Coordinating Principle Investigator/Principle Investigators**	Professor Irene Blackberry<br>Professor Jane Farmer<br>Professor Jennene Greenhill<br>Professor David Perkins<br>Professor Debra Morgan<br>Associate Professor Megan O'Connell<br>Ms Catherine Morley<br>Dr Margaret Winbolt<br>Dr Michael Bauer<br>Dr Clare Wilding
**Research Officers**	Dr Hilary Davis<br>Dr Tshepo Rasekaba<br>Miss Ainsley Robinson<br>Dr Kristina Gottschall<br>Mr Mohammad Hamiduzzaman<br>Ms Kayla Royals

Declaration by Participant

I wish to withdraw from participation in the above research project and understand that such withdrawal will not affect my routine care or my relationships with the researchers.

Name of Participant (please print) ______________________________

Signature ______________________ Date ______________________

In the event that the participant's decision to withdraw is communicated verbally, the Senior Researcher must provide a description of the circumstances below.

Declaration by Researcher

I have given a verbal explanation of the implications of withdrawal from the research project and I believe that the participant has understood that explanation.

Name of Researcher (please print) ______________________________

Signature ______________________ Date ______________________

Note: All parties signing the consent section must date their own signature.
