# Supplementary material for: Web-Based Technologies to Support Carers of People Living With Dementia: Protocol for a Mixed Methods Stepped-Wedge Cluster Randomized Controlled Trial
Source: JMIR Res Protoc. 2022 May 19;11(5):e33023. doi: 10.2196/33023 (PMC9164093; doi:10.2196/33023)
Supplement: Multimedia Appendix 5 [file resprot_v11i5e33023_app5.pdf]

verilyconnect

Verily survey for carers (3)

Welcome

Hello and thank you for your interest in our survey for carers of people with dementia/memory loss as part of the Verily Connect (Virtual Dementia Friendly Rural Communities) project. The Verily Connect project is about using online technologies to increase support for carers of people living with dementia in a rural setting. It is also about helping rural communities to better understand and support people living with dementia, and their family and friends. Verily Connect uses a website and mobile app and video-conferenced support groups as the main ways of increasing support.

A member of our research team should have given you a detailed Participant Information Statement about your involvement in this study and a Participant Identification Number (which you will need to complete this survey). If you haven't received this information, please contact Dr Clare Wilding on c.wilding@latrobe.edu.au or phone: 02 6024 9651.

Your participation in this survey is voluntary; whether or not you complete the survey is entirely your choice. You may skip any questions that you do not feel comfortable to answer. You can stop and exit the survey at any time without giving a reason. However, once you have submitted the survey it will not be possible to withdraw your information.

By completing this survey, you are agreeing to be part of the evaluation of Verily Connect. In public reports about this project, only aggregated information that does not personally identify you will be used.

The project has been approved by the Melbourne Health Human Research Ethics Committee.

If you have any questions about participating in this survey please contact Dr Clare Wilding, John Richards Centre, La Trobe University, PO Box 821, Wodonga, Victoria, 3689 or P: 02 6024 9651 or E: c.wilding@latrobe.edu.au

If you have any complaints or concerns about participating in this survey that the researcher has not been able to answer to your satisfaction, you may contact:

**Reviewing HREC name:**	**Melbourne Health HREC**
**HREC Executive Officer:**	**Manager HREC**
**Telephone:**	**(03) 9342 8530**

Email: **Research@mh.org.au**
Please quote the local reference number: 2017.376

Document identification: 2017.376 Version 2 06.02.2018

verilyconnect

Verily survey for carers (3)

Demographic data

This section asks some basic questions to better understand who is completing this survey.

* 1. Participant identification number

[ ]

2. Do you have a health care card?

- ◯ No
- ◯ Yes

3. Do you have private health insurance?

- ◯ No
- ◯ Yes

4. Do you receive a home care package?

- ◯ No
- ◯ Yes

verilyconnect

Verily survey for carers (3)

Use of dementia services

This section asks about use of services for yourself and/or the person you care for who has cognitive impairment or dementia.

5. Has the person you care for received a diagnosis of dementia or cognitive impairment since you last completed a Verily questionnaire?

- ◯ No
- ◯ Yes, he/she has been diagnosed with cognitive impairment
- ◯ Yes, he/she has been diagnosed with dementia

6. Where does the person you care for live?

- ◯ He/she lives with me
- ◯ He/she lives in the same postcode as me
- ◯ He/she lives 50-100 km from me
- ◯ He/she lives more than 100 km from me

7. Have you or the person you care for received emergency care services (e.g. unexpected visit to hospital) in the last 2 months?

- ◯ No
- ◯ Yes, Please provide details of the situation and services received.

8. What services have you and/or the person you care for used in the past 2 months? (Check all services used)

- [ ] GP
- [ ] Nursing (e.g. district nursing, community nursing)
- [ ] Home help
- [ ] Private domestic services (e.g. private cleaner)
- [ ] Private gardening service
- [ ] Community transport
- [ ] Allied health care (Private or government funded) (e.g. Physiotherapist, Occupational therapist, Speech and Language Therapist, Podiatrist, Psychologist)
- [ ] Day care
- [ ] Respite care
- [ ] Meals on wheels

Other (please specify)

9. Who organised or referred you and/or the person you care for to this service/these services?

10. Do you or the person you care for receive any other kind of support?

- [ ] No
- [ ] Yes, what kind of support and from whom?

11. Are there any other services or support that you or the person you care for need but do not currently have?

- ○ No (skip to Q13)
- ○ Yes

12. What other services or support do you or the person you care for need?

13. In general, how difficult or easy has it been to access services to support you and the person you care for?

very difficult	difficult	neither difficult or easy	easy	very easy
○	○	○	○	○

14. Please rate your local community in relation to each of the following statements:

	Strongly disagree	Disagree	Neither agree nor disagree	Agree	Strongly agree
There is awareness of dementia in my community	○	○	○	○	○
Information about dementia is available in my community	○	○	○	○	○
Social activities for people with dementia are available in my community	○	○	○	○	○
The physical environment allows people with dementia to move about safely and easily	○	○	○	○	○
Affordable, accessible transport is available in my community	○	○	○	○	○
Healthcare is easily accessible in my community	○	○	○	○	○
Our community has support groups for people with dementia and cognitive impairment	○	○	○	○	○
Our community has support groups for carers in the community	○	○	○	○	○
Our community has groups and activities for people with dementia and cognitive impairment and the people who care for them to attend together	○	○	○	○	○

verilyconnect

Verily survey for carers (3)

Usage of and familiarity with technology

15. Do you have a landline telephone?

- ◯ No
- ◯ Yes

16. Do you have a mobile phone?

- ◯ No
- ◯ Yes - I have a standard mobile phone for calls and texts
- ◯ Yes - I have a smartphone with Internet access

17. Do you have an electronic tablet device (e.g. iPad)?

- ◯ No
- ◯ Yes, iPad
- ◯ Yes, Android
- ◯ Yes, Windows or another platform

18. Have you used a computer in the past month?

- ◯ No
- ◯ Yes, I used my home computer
- ◯ Yes, I used a computer outside my home (e.g. at library or community centre)

19. Have you sent messages by e-mail or text message within the past month?

- Never
- Rarely
- Some days
- Most days

20. Have you used social media in the past month? (Check all social media types used)

- I have not used social media
- Facebook
- Twitter
- Skype
- Instagram
- Linked In
- Google Hangouts

Other (please specify)

21. Have you gone on the Internet or online to do the following in the past month:

	Yes	No
Shop for groceries or personal items		
Pay bills or do banking		
Contact or find any health care provider		
Manage Medicare or health insurance		
Get information about health conditions		
Use the My Aged Care website		
Communicate with others (e.g. by Skype or social media)		

22. Do you have Internet access at home?

- ◯ No
- ◯ Yes, wi-fi
- ◯ Yes, broadband
- ◯ Yes, other type of access

23. Do you have problems with lack of mobile phone service in your home? (e.g. a "blackspot" in which you cannot connect online by mobile phone or tablet)

- ◯ No, I can easily connect via mobile
- ◯ Yes, I sometimes have problems with mobile coverage
- ◯ Yes, I consistently have problems with mobile coverage

verilyconnect

Verily survey for carers (3)

Social connections

24. Which image best represents how close or connected you feel to other carers of people with dementia? (Dark circle is you and light circle is other carers of people with dementia)

- ◯ image 1
- ◯ image 2
- ◯ image 3
- ◯ image 4
- ◯ image 5
- ◯ image 6
- ◯ image 7

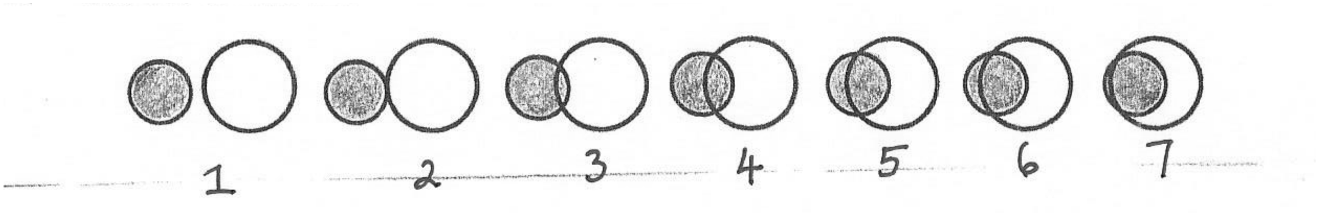

25. Which image best represents how close or connected you feel to service providers? (Dark circle is you and light circle is service providers)

- ◯ image 1
- ◯ image 2
- ◯ image 3
- ◯ image 4
- ◯ image 5
- ◯ image 6
- ◯ image 7

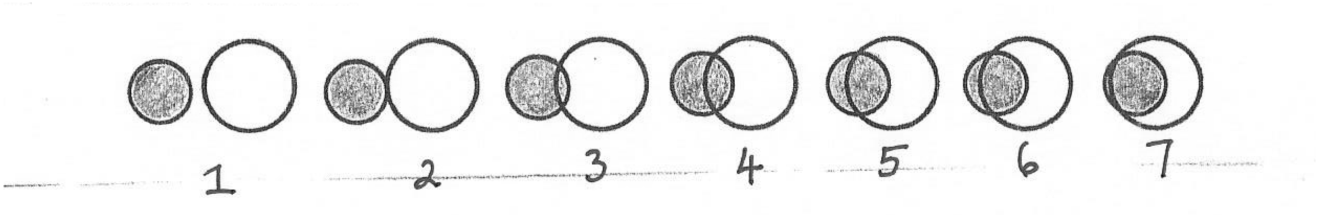

verilyconnect

Verily survey for carers (3)

Challenges of caring

Caring for someone with dementia or cognitive impairment can be challenging. Your answers to questions in this section helps us to understand how the caring experience is impacting on you and your life.

The following is a list of statements which reflect how people sometimes feel when taking care of another person. After each statement, indicate how often you feel that way: never, rarely, sometimes, quite frequently, or nearly always. There are no right or wrong answers.

"Your relative" refers to the person you are caring for with dementia or cognitive impairment.

26. Do you feel that your relative asks for more help than he or she needs?

◯ Never ◯ Rarely ◯ Sometimes ◯ Quite frequently ◯ Nearly always

27. Do you feel that because of the time you spend with your relative you don't have enough time for yourself?

◯ Never ◯ Rarely ◯ Sometimes ◯ Quite frequently ◯ Nearly always

28. Do you feel stressed between caring for your relative and trying to meet other responsibilities for your family or work?

◯ Never ◯ Rarely ◯ Sometimes ◯ Quite frequently ◯ Nearly always

29. Do you feel embarrassed about your relative's behaviour?

◯ Never ◯ Rarely ◯ Sometimes ◯ Quite frequently ◯ Nearly always

30. Do you feel angry when you are around your relative?

◯ Never ◯ Rarely ◯ Sometimes ◯ Quite frequently ◯ Nearly always

31. Do you feel that your relative currently affects your relationship with other family members?

◯ Never ◯ Rarely ◯ Sometimes ◯ Quite frequently ◯ Nearly always

32. Are you afraid about what the future holds for your relative?

◯ Never ◯ Rarely ◯ Sometimes ◯ Quite frequently ◯ Nearly always

33. Do you feel that your relative is dependent upon you?

◯ Never ◯ Rarely ◯ Sometimes ◯ Quite frequently ◯ Nearly always

34. Do you feel strained when you are around your relative?

◯ Never ◯ Rarely ◯ Sometimes ◯ Quite frequently ◯ Nearly always

35. Do you feel that your health has suffered because of your involvement with your relative?

◯ Never ◯ Rarely ◯ Sometimes ◯ Quite frequently ◯ Nearly always

36. Do you feel that you don't have as much privacy as you would like, because of your relative?

◯ Never ◯ Rarely ◯ Sometimes ◯ Quite frequently ◯ Nearly always

37. Do you feel that your social life has suffered because you are caring for your relative?

◯ Never ◯ Rarely ◯ Sometimes ◯ Quite frequently ◯ Nearly always

38. Do you feel uncomfortable having your friends over because of your relative?

◯ Never ◯ Rarely ◯ Sometimes ◯ Quite frequently ◯ Nearly always

39. Do you feel that your relative seems to expect you to take care of him or her, as if you were the only one he or she could depend on?

◯ Never ◯ Rarely ◯ Sometimes ◯ Quite frequently ◯ Nearly always

40. Do you feel that you don't have enough money to care for your relative, in addition to the rest of your expenses?

◯ Never ◯ Rarely ◯ Sometimes ◯ Quite frequently ◯ Nearly always

41. Do you feel that you will be unable to take care of your relative much longer?

◯ Never ◯ Rarely ◯ Sometimes ◯ Quite frequently ◯ Nearly always

42. Do you feel that you have lost control of your life since your relative's illness?

◯ Never ◯ Rarely ◯ Sometimes ◯ Quite frequently ◯ Nearly always

43. Do you wish that you could just leave the care of your relative to someone else?

◯ Never ◯ Rarely ◯ Sometimes ◯ Quite frequently ◯ Nearly always

44. Do you feel uncertain about what to do about your relative?

◯ Never ◯ Rarely ◯ Sometimes ◯ Quite frequently ◯ Nearly always

45. Do you feel that you should be doing more for your relative?

◯ Never ◯ Rarely ◯ Sometimes ◯ Quite frequently ◯ Nearly always

46. Do you feel that you could do a better job in caring for your relative?

◯ Never ◯ Rarely ◯ Sometimes ◯ Quite frequently ◯ Nearly always

47. Overall, how burdened do you feel in caring for your relative?

○ Not at all ○ A little ○ Moderately ○ Quite a bit ○ Extremely

verilyconnect

Verily survey for carers (3)

Social Support Survey

48. How often is each of the following kinds of support available to you if you need it?

	None of the time	A little of the time	Some of the time	Most of the time	All of the time
Someone you can count on to listen to you when you need to talk	○	○	○	○	○
Someone to give you information to help you understand a situation	○	○	○	○	○
Someone to give you good advice about a crisis	○	○	○	○	○
Someone to confide in or talk to about yourself or your problems	○	○	○	○	○
Someone whose advice you really want	○	○	○	○	○
Someone to share your most private worries and fears with	○	○	○	○	○
Someone to turn to for suggestions about how to deal with a personal problem	○	○	○	○	○
Someone who understands your problems	○	○	○	○	○
Someone to help you if you were confined to a bed	○	○	○	○	○

	None of the time	A little of the time	Some of the time	Most of the time	All of the time
Someone to take you to the doctor if you needed it	○	○	○	○	○
Someone to prepare your meals if you were unable to do it yourself	○	○	○	○	○
Someone to help with daily chores if you were sick	○	○	○	○	○
Someone who shows you love and affection	○	○	○	○	○
Someone to love and make you feel wanted	○	○	○	○	○
Someone who hugs you	○	○	○	○	○
Someone to have a good time with	○	○	○	○	○
Someone to get together with for relaxation	○	○	○	○	○
Someone to do something enjoyable with	○	○	○	○	○
Someone to do things with you to get your mind off things	○	○	○	○	○
