# Supplementary material for: Web-Based Technologies to Support Carers of People Living With Dementia: Protocol for a Mixed Methods Stepped-Wedge Cluster Randomized Controlled Trial
Source: JMIR Res Protoc. 2022 May 19;11(5):e33023. doi: 10.2196/33023 (PMC9164093; doi:10.2196/33023)
Supplement: Multimedia Appendix 6 [file resprot_v11i5e33023_app6.docx]

**Volunteer Focus Group Guide**

1. Please tell us a little bit about the activities you completed as a volunteer in the VERILY project

2. What was good or interesting about being a volunteer in VERILY?

3. What was challenging or difficult about being a volunteer in VERILY?

4. What parts of VERILY do you think worked well?

5. What parts of VERILY could be improved?

6. Would you recommend carers to get involved in VERILY activities? Please talk a bit about why you would recommend or not recommend VERILY

7. Would you recommend others to be a volunteer in VERILY? Please talk a bit about why you would recommend or not recommend volunteering in VERILY

8. Is there anything else you would like to say or anything else you think is important for us to know?

Thank you
