# Supplementary material for: Web-Based Technologies to Support Carers of People Living With Dementia: Protocol for a Mixed Methods Stepped-Wedge Cluster Randomized Controlled Trial
Source: JMIR Res Protoc. 2022 May 19;11(5):e33023. doi: 10.2196/33023 (PMC9164093; doi:10.2196/33023)
Supplement: Multimedia Appendix 7 [file resprot_v11i5e33023_app7.docx]

# Interview guide for all participants (September 2019)

1. Have you used the Verily Connect app?
   1. (if Yes) What have you used the app for? How often have you used the app? What features worked well and what did not work? How can we improve the app?
   2. (if No) How have you sourced the information you need? How have you met your (support) needs? Do you have any suggestions about how we can assist people to use the app?
2. (Carers only) Have you continued to attend the Zoom peer support meetings?
3. (Volunteers only) Please tell me about any Verily Connect volunteering activities you have completed over the past 5 months. Please tell me about any other volunteering activities you have completed over the past 5 months.
4. (Staff only) Please tell me about any occasions during which you have discussed or recommended the Verily Connect app to others (e.g. clients/customers, colleagues, community organisations).
5. Do you have any other feedback about or suggestions for Verily Connect?
6. Do you have any other questions or comments?

Thank you for participating in the VERILY project.
