# Supplementary material for: Web-Based Technologies to Support Carers of People Living With Dementia: Protocol for a Mixed Methods Stepped-Wedge Cluster Randomized Controlled Trial
Source: JMIR Res Protoc. 2022 May 19;11(5):e33023. doi: 10.2196/33023 (PMC9164093; doi:10.2196/33023)
Supplement: Multimedia Appendix 8 [file resprot_v11i5e33023_app8.docx]

**Interview guide for staff**

(Using Consolidated Framework for Implementation Research Questions, available at: <https://cfirguide.org/guide/app/#/guide_select>)

1. Please tell me about the activities you participated in as part of the VERILY project
2. How complicated was it to implement VERILY? Were changes or alterations made to the way that VERILY was implemented in your community?
3. What other carer support programs are available in your area? How does VERILY compare to other existing programs; does it replace or complement other programs? Is there another way of attaining support that carers would rather use? Do you think there was a strong need for VERILY?
4. How well do you think VERILY met the needs of your clients? Do you think VERILY was effective in your area?
5. What barriers did your clients experience with accessing VERILY?
6. What stories have you heard about the experiences of participants with VERILY?
7. How do you think your organisation’s culture (general beliefs, values, assumptions that people embrace) affected the implementation of VERILY? What was the general level of receptivity in your organisation to implementing VERILY?
8. How essential is VERILY to assisting you to meet the needs of your clients or your other organisational goals? Please describe the activities or initiatives that have the highest priority for your organisation? To what extent did VERILY take a backseat to other high-priority initiatives? How did you juggle completing priorities in your work?
9. To what extent does implementing VERILY provide an advantage to your town compared to other towns?
10. How well did VERILY fit with existing work processes and practices in your area? Can you describe how VERILY will be integrated into your ongoing work processes?
11. Did you have sufficient resources to implement VERILY? If yes, What was the quality of these resources?
12. What training did you receive in implementing VERILY? How prepared did you feel to use VERILY initiatives? Who did you ask if you had questions about VERILY or its implementation?
13. Were there incentives to help you ensure that the implementation of VERILY was successful?
14. What level of endorsement or support for VERILY did you see or hear from leaders? What level of involvement did leadership at your organisation have with VERILY? What kind of support or actions from leaders was needed to help VERILY be implemented?
15. Did you or others act as champions for VERILY? If yes, What behaviours or actions did you or others take to champion VERILY? What steps have been taken to encourage people to commit to using VERILY? What communication strategy was used to get the word out about VERILY?
16. Is there anything else that you would like to say about VERILY that we haven’t covered?
